# *T*_1_ mapping performance and measurement repeatability: results from the multi-national *T*_1_ mapping standardization phantom program (T1MES)

**DOI:** 10.1186/s12968-020-00613-3

**Published:** 2020-05-07

**Authors:** Gabriella Captur, Abhiyan Bhandari, Rüdiger Brühl, Bernd Ittermann, Kathryn E. Keenan, Ye Yang, Richard J. Eames, Giulia Benedetti, Camilla Torlasco, Lewis Ricketts, Redha Boubertakh, Nasri Fatih, John P. Greenwood, Leonie E. M. Paulis, Chris B. Lawton, Chiara Bucciarelli-Ducci, Hildo J. Lamb, Richard Steeds, Steve W. Leung, Colin Berry, Sinitsyn Valentin, Andrew Flett, Charlotte de Lange, Francesco DeCobelli, Magalie Viallon, Pierre Croisille, David M. Higgins, Andreas Greiser, Wenjie Pang, Christian Hamilton-Craig, Wendy E. Strugnell, Tom Dresselaers, Andrea Barison, Dana Dawson, Andrew J. Taylor, François-Pierre Mongeon, Sven Plein, Daniel Messroghli, Mouaz Al-Mallah, Stuart M. Grieve, Massimo Lombardi, Jihye Jang, Michael Salerno, Nish Chaturvedi, Peter Kellman, David A. Bluemke, Reza Nezafat, Peter Gatehouse, James C. Moon, on behalf of the T1MES Consortium

**Affiliations:** 1 UCL Institute of Cardiovascular Science, University College London, Gower Street, London, WC1E 6BT UK; 2 UCL MRC Unit for Lifelong Health and Ageing, University College London, 1-19 Torrington Place, London, WC1E 7BH UK; 3 Cardiology Department, The Royal Free Hospital, Centre for Inherited Heart Muscle Conditions, Pond Street, Hampstead, London, NW3 2QG UK; 4 UCL Medical School, University College London, Bloomsbury Campus, Gower Street, London, WC1E 6BT UK; 5 Physikalisch-Technische Bundesanstalt (PTB), Abbestr. 2–12, D-10587 Berlin, Germany; 6 National Institute of Standards and Technology (NIST), Boulder, MS 818.03, 325 Broadway, Boulder, CO USA; 7 Department of Cardiology, Sir Run Run Shaw Hospital, Zhejiang University, Hangzhou, 310016 Zhejiang People’s Republic of China; 8 Department of Physics, Imperial College London, Prince Consort Rd, London, SW7 2BB UK; 9 Department of Radiology, Guys and St Thomas NHS Foundation Trust, London, UK; 10 University of Milan-Bicocca, Piazza dell’Ateneo Nuovo 1, 20100 Milan, Italy; 11 Cardiovascular Biomedical Research Unit, Queen Mary University of London, London, E1 4NS UK; 12 Multidisciplinary Cardiovascular Research Center & Division of Biomedical Imaging, Leeds Institute of Cardiovascular and Metabolic Medicine, University of Leeds, Leeds, UK; 13 Department of Radiology & Nuclear Medicine, Maastricht University Medical Centre, PO Box 5800, 6202 AZ Maastricht, The Netherlands; 14 Bristol Heart Institute, National Institute of Health Research (NIHR) Biomedical Research Centre, University Hospitals Bristol NHS Foundation Trust and University of Bristol, Upper Maudlin St, Bristol, BS2 8HW UK; 15 Department of Radiology, Leiden University Medical Centre, Albinusdreef 2, 2333 ZA Leiden, The Netherlands; 16 University Hospitals Birmingham NHS Foundation Trust, Edgbaston, Birmingham, B15 2TH UK; 17 UK Albert B. Chandler Hospital - Pavilion G, Gill Heart & Vascular Institute, Lexington, KY 40536 USA; 18 Institute of Cardiovascular and Medical Sciences, RC309 Level C3, Bhf Gcrc, Glasgow, Scotland G12 8TA UK; 19 Department of Multidisciplinary Clinical Studies, Lomonosov Moscow State University, Moscow, Russia; 20 University Hospital Southampton Foundation Trust, Tremona Road, Southampton, Hampshire SO16 6YD UK; 21 Department of Radiology and Nuclear Medicine, Oslo University Hospital, Sognsvannsveien 20, 0372 Oslo, Norway; 22 San Raffaele Hospital, Via Olgettina 60, 20132 Milan, Italy; 23 INSA, CNRS UMR 5520, INSERM U1206, University of Lyon, UJM-Saint-Etienne, CREATIS, F-42023 Saint-Etienne, France; 24 Department of Radiology, University Hospital Saint-Etienne, Saint-Etienne, France; 25 Philips, Philips Centre, Unit 3, Guildford Business Park, Guildford, Surrey GU2 8XG UK; 26 SiemensHealthcare GmbH, Erlangen, Germany; 27 Resonance Health, 278 Stirling Highway, Claremont, WA 6010 Australia; 28 The Prince Charles Hospital, Griffith University and University of Queensland, Brisbane, Australia; 29 Department of Radiology, Universitair Ziekenhuis Leuven, Leuven, UZ Belgium; 30 Fondazione Toscana Gabriele Monasterio, Pisa, Italy; 31 School of Medicine and Dentistry, University of Aberdeen, Polwarth Building, Foresterhill, Aberdeen, AB25 2ZD Scotland, UK; 32 Department of Cardiovascular Medicine, Alfred Hospital, Melbourne, Australia; 33 Baker Heart and Diabetes Institute, Melbourne, Australia; 34 Department of Medicine, Monash University, Melbourne, Australia; 35 Department of Medicine, Montreal Heart Institute and Université de Montréal, 5000 Bélanger Street, Montreal, QC H1T 1C8 Canada; 36 Department of Internal Medicine – Cardiology, Deutsches Herzzentrum Berlin, Berlin, Germany; 37 Department of Internal Medicine and Cardiology, Charité - Universitätsmedizin Berlin, Campus Virchow Klinikum, Berlin, Germany; 38 King Abdulaziz Cardiac Center (KACC) (Riyadh), National Guard Health Affairs, Riyadh, Kingdom of Saudi Arabia; 39 The University of Sydney School of Medicine, Camperdown, NSW 2006 Australia; 40 I.R.C.C.S., Policlinico San Donato, Piazza Edmondo Malan, 2, 20097 San Donato Milanese, MI Italy; 41 Department of Medicine (Cardiovascular Division), Beth Israel Deaconess Medical Center, Harvard Medical School, Cardiology East Campus, Room E/SH455, 330 Brookline Ave, Boston, MA 02215 USA; 42 University of Virginia Health System, 1215 Lee St, PO Box 800158, Charlottesville, VA 22908 USA; 43 National Heart, Lung, and Blood Institute, National Institutes of Health, Bethesda, MD 20892-1061 USA; 44 Department of Radiology, University of Wisconsin School of Medicine and Public Health, Madison, WI 53792-3252 USA; 45 CMRI Department, Royal Brompton Hospital, Sydney Street, London, SW3 6NP UK; 46 Barts Heart Center, St Bartholomew’s Hospital, West Smithfield, London, EC1A 7BE UK

**Keywords:** *T*_1_ mapping, Standardization, Calibration, Phantom, Repeatability, Extracellular volume

## Abstract

**Background:**

The *T*_1_ Mapping and Extracellular volume (ECV) Standardization (T1MES) program explored *T*_1_ mapping quality assurance using a purpose-developed phantom with Food and Drug Administration (FDA) and Conformité Européenne (CE) regulatory clearance. We report *T*_1_ measurement repeatability across centers describing sequence, magnet, and vendor performance.

**Methods:**

Phantoms batch-manufactured in August 2015 underwent 2 years of structural imaging, *B*_0_ and *B*_1_, and “reference” slow *T*_1_ testing. Temperature dependency was evaluated by the United States National Institute of Standards and Technology and by the German Physikalisch-Technische Bundesanstalt. Center-specific *T*_1_ mapping repeatability (maximum one scan per week to minimum one per quarter year) was assessed over mean 358 (maximum 1161) days on 34 1.5 T and 22 3 T magnets using multiple *T*_1_ mapping sequences. Image and temperature data were analyzed semi-automatically. Repeatability of serial *T*_1_ was evaluated in terms of coefficient of variation (CoV), and linear mixed models were constructed to study the interplay of some of the known sources of *T*_1_ variation.

**Results:**

Over 2 years, phantom gel integrity remained intact (no rips/tears), *B*_0_ and *B*_1_ homogenous, and “reference” *T*_1_ stable compared to baseline (% change at 1.5 T, 1.95 ± 1.39%; 3 T, 2.22 ± 1.44%). Per degrees Celsius, 1.5 T, *T*_1_ (MOLLI 5s(3s)3s) increased by 11.4 ms in long native blood tubes and decreased by 1.2 ms in short post-contrast myocardium tubes. Agreement of estimated *T*_1_ times with “reference” *T*_1_ was similar across Siemens and Philips CMR systems at both field strengths (adjusted *R*^2^ ranges for both field strengths, 0.99–1.00). Over 1 year, many 1.5 T and 3 T sequences/magnets were repeatable with mean CoVs < 1 and 2% respectively. Repeatability was narrower for 1.5 T over 3 T. Within T1MES repeatability for native *T*_1_ was narrow for several sequences, for example, at 1.5 T, Siemens MOLLI 5s(3s)3s prototype number 448B (mean CoV = 0.27%) and Philips modified Look-Locker inversion recovery (MOLLI) 3s(3s)5s (CoV 0.54%), and at 3 T, Philips MOLLI 3b(3s)5b (CoV 0.33%) and Siemens shortened MOLLI (ShMOLLI) prototype 780C (CoV 0.69%). After adjusting for temperature and field strength, it was found that the *T*_1_ mapping sequence and scanner software version (both *P* < 0.001 at 1.5 T and 3 T), and to a lesser extent the scanner model (*P* = 0.011, 1.5 T only), had the greatest influence on *T*_1_ across multiple centers.

**Conclusion:**

The T1MES CE/FDA approved phantom is a robust quality assurance device. In a multi-center setting, *T*_1_ mapping had performance differences between field strengths, sequences, scanner software versions, and manufacturers. However, several specific combinations of field strength, sequence, and scanner are highly repeatable, and thus, have potential to provide standardized assessment of *T*_1_ times for clinical use, although temperature correction is required for native *T*_1_ tubes at least.

## Introduction

*T*_1_ mapping aids clinicians in the assessment and diagnosis of myocardial disease. However, measurement needs to be stable over time with transferable values. Knowledge of normal reference ranges would benefit from not requiring local healthy subject scanning, and the pooling of multi-scanner datasets would have advantages such as increasing available sample sizes for the detection of subtle effects or subgroup analysis and increasing result robustness and generalizability, lowering the chance of unforeseen bias when compared to single-center data [1]. Combining results however introduces sequence, magnet, and field strength bias [2]. The field of *T*_1_ mapping would therefore benefit from a “*T*_1_ standard” to enable cross-center *T*_1_ mapping data pooling and delivery [3]—like the international normalized ratio (INR) which makes it possible to adjust the dosing of vitamin K antagonists regardless of which laboratory has performed the test [4].

The *T*_1_ Mapping and Extracellular volume (ECV) Standardization (T1MES) phantom program was established to explore *T*_1_ mapping quality assurance at 1.5 T and 3 T and understand the feasibility of delivering a “*T*_1_ standard” [5]. The first step toward the goal was development and mass-production of a phantom [5] and its European Union Conformité Européenne (CE) and United States Food and Drug Administration (FDA) regulatory clearance. In September 2015, cardiovascular magnetic resonance (CMR) centers worldwide joined the T1MES consortium and committed to submit a minimum of 12 months of center-specific *T*_1_ mapping data. Data submitted have now been analyzed to explore phantom performance.

We report phantom data at 1 and 2 years using various *T*_1_ mapping sequences, temperature sensitivity, and include platform performance, although we emphasize that comparison of different *T*_1_ methods and systems was not the main aim, rather investigating long-term stability towards the “*T*_1_ standard”. This involved modeling some of the potential sources of the *T*_*1*_ variation longitudinally and between T1MES centers to identify the most influential factors.

## Methods

**Fig. 1:**
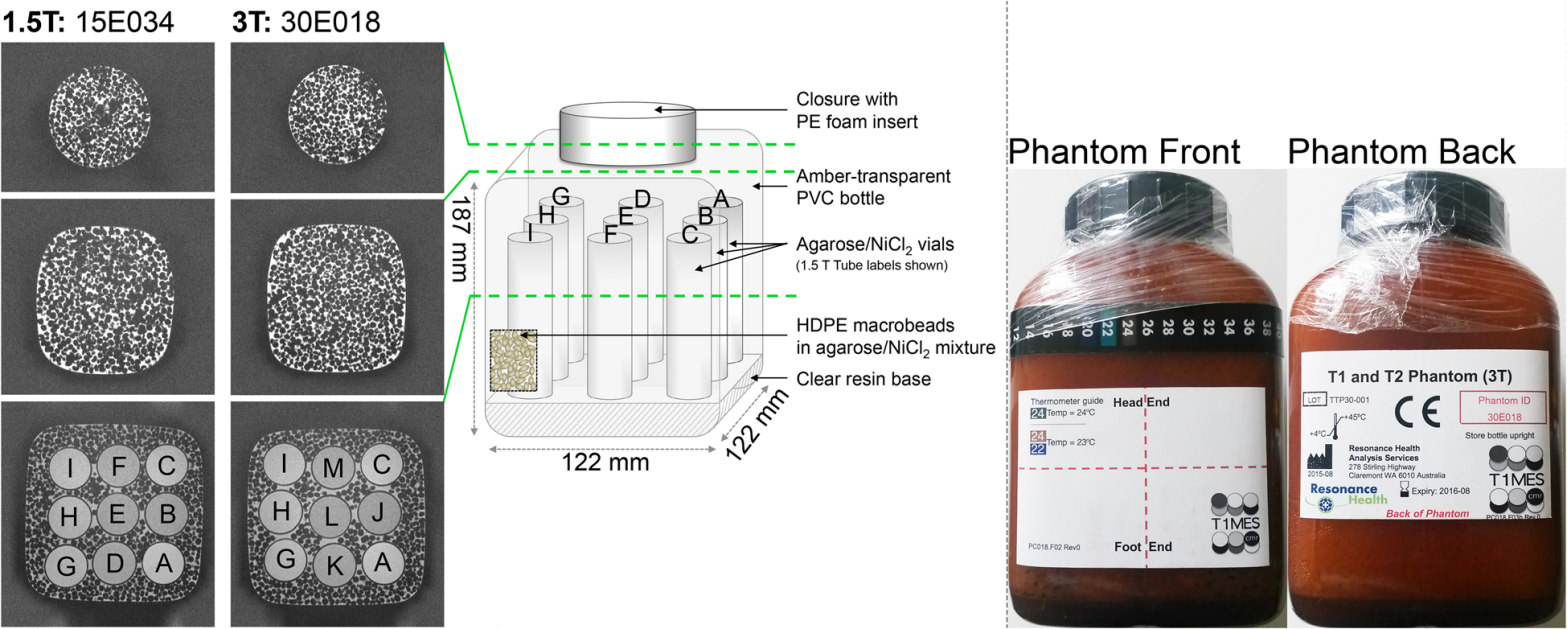
Left panel: Exemplar high-resolution (0.42 mm isotropic) imaging conducted at 3 T on 5 bottles (15E031; 15E033; 15E034 shown here; 30E017; 30E018 shown here) sampled out of the original batch in August 2017 (this is 2 years post manufacture) confirmed their structural integrity. The whole length of the phantom was imaged (three exemplar slices only shown here, represented by the green dashed lines). The internal tubes are labeled. Surrounding the tubes is a speckled pattern due to high density polyethylene (HDPE) macrobeads in an agarose/NiCl_2_ mixture . Confluent bright patches between tubes represent patches of agarose/NiCl_2_ mixture due to displacement of macrobeads. There were no signs of structural deterioration of the phantoms 2 years after manufacture. Middle panel: The nine tubes are supported on a translucent resin base composed of unsaturated polyester/styrene. The matrix fill is packed with compact HDPE pellets and agarose/NiCl_2_ mixture. Right panel: The outer physical appearance (front and back surfaces) of a phantom (30E018) at 2 years post manufacture (the plastic packaging wrap around the bottle cap dates back to the time of manufacture). HDPE, high-density polyethylene; NiCl_2_, nickel chloride; PE, polyethylene; PVC, polyvinyl chloride

The development and description of the T1MES phantom (Fig. 1) has been previously reported [5]. Briefly, the T1MES phantom was designed to be field-strength specific (i.e., separate 1.5 T and 3 T models). Each phantom contains four tubes representing human native blood/myocardial *T*_1_ and *T*_2_ values (i.e., pre-gadolinium-based contrast agent [GBCA] values) and five tubes representing human post-GBCA blood/myocardial values. While the main aim of the present study was the collection and analysis of the multi-center data (see the “Methods part 2—Multi-center phantom testing” section), some other tests were applied to a small number of the phantoms during the 2 years to explore the utility of T1MES as a quality assurance device, and these tests are described here first (“Methods part 1—Evaluation of the phantom”). Imaging biomarker terms used follow the recommendations of the Quantitative Imaging Biomarkers Alliance (QIBA) of the Radiological Society of North America (RSNA) [6].

### Methods part 1—Evaluation of the phantom

#### Structural integrity

Gel integrity and aging were checked at each submission time point for participating sites through the manual inspection of localizers that formed part of the minimum dataset requirement for participation. In addition, a high-resolution, isotropic, three-dimensional (3D) gradient echo sequence (0.42 mm^3^) was run on four phantoms (three 1.5 T phantoms; one 3 T phantom) at baseline (October 2015) and at 2 years post manufacturing in each case using a 3 T MAGNETOM Skyra (Siemens Healthineers, Erlangen, Germany; software syngo MR D13C). The sequence acquired two overlapping slabs (due to scanner software constraints), each with two directions of phase encoding, a slow repetition time (repetition time, TR = 17 ms), and narrow sampling bandwidth (250 Hz/pixel) for better signal-to-noise ratio (SNR). This sequence had weak *T*_1_ and *T*_2_ image contrast and was only for structural examination.

#### “Reference” *rT*_1_ and *rT*_2_ data

Baseline (October 2015) “reference” *T*_1_ and *T*_2_ values (*rT*_1_, *rT*_2_) were acquired at the Royal Brompton Hospital CMR Unit using basic single-slice TR = 10 s inversion recovery spin echo (IRSE, 8 inversion times [TI] from 25 to 3200 ms) and single-slice repetition time (TR) = 10 s SE (8 echo times [TE] from 10 to 640 ms) [5] respectively. These sequences were identically repeated at 2 years on the same three 1.5 T phantoms and on the same three 3 T phantoms sampled from the production batch. The identifying serial numbers of the three 1.5 T phantoms were 15E031, 15E033, and 15E034, and these phantoms were scanned on a 1.5 T MAGNETOM Avanto [Siemens Healthineers; software syngo MR B17A]. The three 3 T phantoms were 30E001, 30E017, and 30E018, and they were scanned on a 3 T MAGNETOM Skyra [Siemens Healthineers; software syngo MR D13C].

Separate *rT*_1_ and *rT*_2_ data were acquired on the same 3 T phantom (30E021) at the German National Metrology Institute, Physikalisch-Technische Bundesanstalt (PTB) over a period of 1041 days (64 scans) commencing September 2015 (3 T MAGNETOM Verio (Siemens Healthineers; software syngo MR B17A). Sequences used for *rT*_1_ and *rT*_2_ were respectively basic single-slice TR = 8000 ms IRSE (IRSE, 7 TI from 25 to 4800 ms) and single-slice TR = 3000 ms SE (5 TE from 24 to 400 ms).

#### Temperature sensitivity

**Fig. 2:**
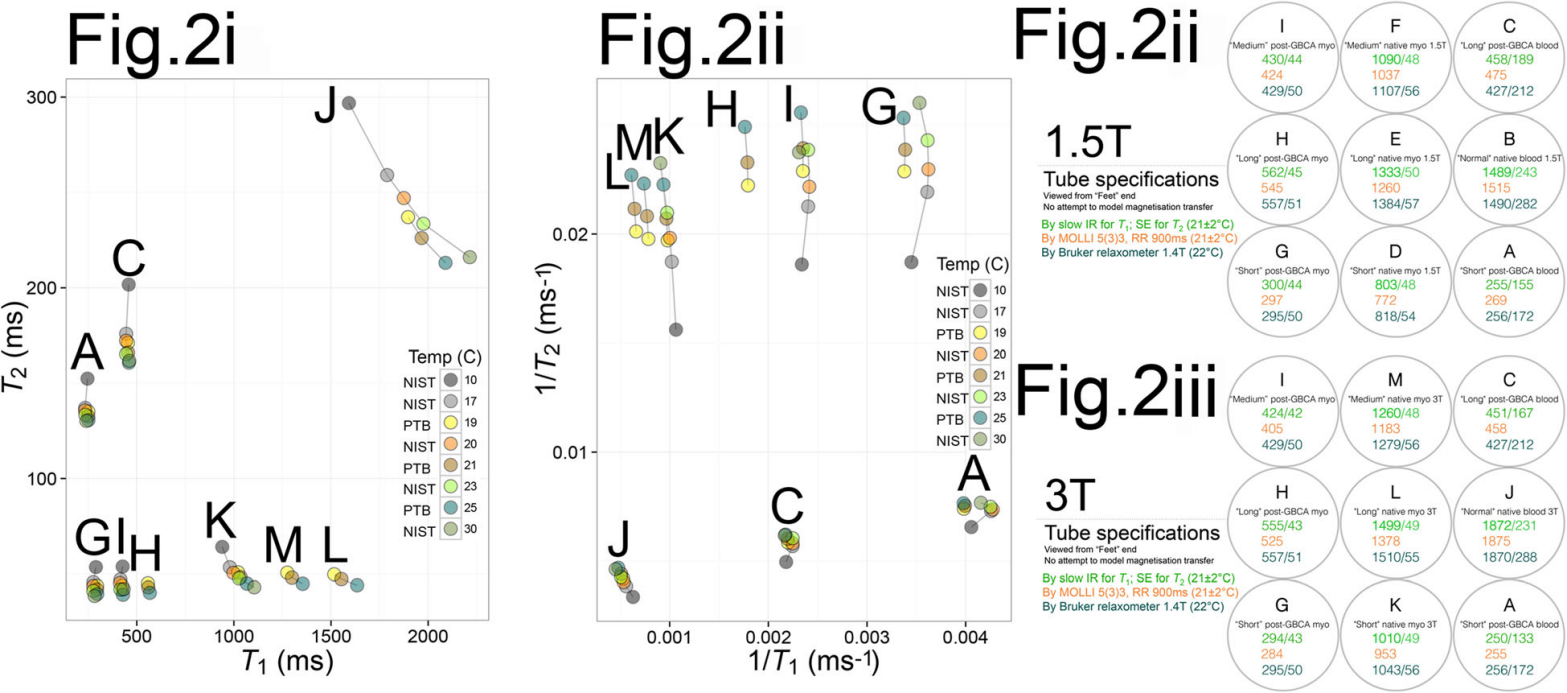
Temperature experiments (**i**) performed at two national metrology institutes: the US National Institute of Standards and Technology (NIST) laboratory after 1 year on six loose tubes from T1MES and at Physikalisch-Technische Bundesanstalt (PTB) on phantom 30E012. **ii** and **iii** indicate to which field-strength device the tested tubes belong. At NIST, *T*_1_ and *T*_2_ were measured on a Varian/Agilent small bore scanner operating at 1.5 T in a temperature-controlled environment. Temperatures were measured using a fiber optic probe. At PTB, *T*_1_ and *T*_2_ were measured on a 3 T MAGNETOM Verio scanner (Siemens Healthineers; software syngo MR B17A). The phantom was always stored, moved, and scanned while resting in a Styrofoam box to ensure that the temperatures picked at bottle hull reflects the tube temperature. At scan time, the box was placed in the head coil (12 ch) of the PTB 3 T scanner. Temperatures were measured using a Pt100 resistance thermometer. Similar to temperature dependency results immediately after phantom manufacture, [5] short-*T*_1_ tubes (modeling post-GBCA myocardium and blood) are more stable with temperature than very long-*T*_1_ tubes (native blood) where *T*_1_ increases more significantly with temperature

The following three methods were used:

First, controlled-temperature experiments over the range 10–30 °C were conducted at the United States National Institute of Standards and Technology (NIST) on six loose T1MES tubes at 1 year (Fig. 2i). *T*_1_ and *T*_2_ were measured at 10, 17, 20, 23, and 30 °C on an VnmrJ4 small-bore scanner operating at 1.5 T (Varian Medical Systems, Palo Alto, California, USA) in a temperature-controlled environment using a fiber optic temperature probe. *T*_1_ was measured by IRSE (TR = 10 s, TI = 50–3000 ms) and *T*_2_ by SE (TR = 10 s, TE = 15–960 ms).

Second, controlled-temperature experiments at 19, 21, and 25 °C were conducted at the PTB laboratory on T1MES phantom 30E012 at 1 year (also Fig. 2i). *T*_1_ and *T*_2_ were measured on a 3 T MAGNETOM Verio scanner (Siemens Healthineers; software syngo MR B17A) using a Pt100 resistance thermometer. *T*_1_ was measured by IRSE (TR = 8000 ms, TI = 25–4800 ms) and *T*_2_ by SE (TR = 3000 ms, TE = 24–400 ms).

Third, for each T1MES phantom scan at all centers, temperature was measured using liquid crystal thermometers adhered to every phantom. These measurements were pooled and analyzed to derive temperature-correction algorithms (see Statistical Analysis).

#### *B*_0_ and *B*_1_ uniformity

These uniformities and the fundamental distortion of *B*_1_ by water dielectric permittivity especially at 3 T had been tested at baseline (October 2015, previously reported [5]). These uniformities were mapped later to check against “cracking” of the gel and subsequent impact of air gaps on *B*_0_ in particular, while potential “clumping” of the plastic beads over time might in theory affect the *B*_1_ [5]. We therefore considered it prudent to check whether anything unexpected occurred over the long term.

*B*_0_ uniformity was therefore mapped at 2 years in six phantoms, in the transverse slice, midway along the length of the tubes, using a multi-echo gradient echo sequence, based on the phase difference between known TEs [7]. A frequency range of ± 50 Hz across the phantom was considered acceptable, based on published *T*_1_ mapping off-resonance sensitivity [8]. *B*_1_ homogeneity was similarly evaluated using flip angle (FA) maps (double angle method using FA 60° and 120° [θ1, 2 × θ1] with long TR [8 s], and 4 ms sinc [− 3π to + 3π] slice excitation profiles to minimize error due to FA variation through the slice).

### Methods part 2—Multi-center phantom testing

#### Serial, multi-center *T*_1_ mapping data

**Fig. 3:**
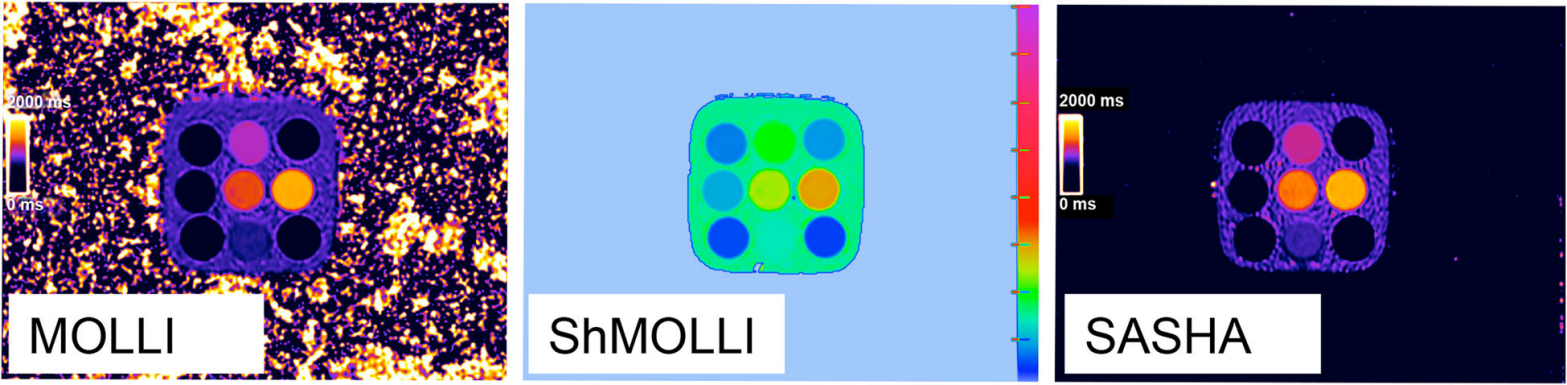
Representative maps exactly as they were submitted by collaborating sites, showing the 3 commonly used *T*_1_ mapping sequences appraised in T1MES: MOLLI 5s(3s)3s [448B], ShMOLLI 5b(1b)1b(1b)1b [1041B], and SASHA VE 11A

The T1MES user manual (10.6084/m9.figshare.c.3610175_D1.v1) defined strict scanning instructions (scanning and shim volume strictly at isocenter, use of same supporting materials, etc.). Each contributed T1MES dataset (localizers, sets of inversion recovery images, and inline scanner-generated *T*_1_ maps, Fig. 3) underwent initial quality assurance, checking orientation, and isocenter (through visual inspection of localizers and maps and semi-automatically by inspecting metadata contained in Digital Imaging and COmmunications in Medicine [DICOM] headers “ImagePositionPatient” and “ImageOrientationPatient”) and to exclude image artifacts. All Siemens sequences except MyoMaps product variant and all Philips (Philips Healthcare, Best, the Netherlands) sequences except CardiacQuant product variant were prototypes. Any tubes with artifacts detected by operator inspection of the submitted *T*_1_ maps were excluded from the analysis. Software version changes were captured automatically from DICOM headers (“StationName” and “SoftwareVersion”).

The *T*_1_ measurements from T1MES datasets (directly using only the parametric maps submitted, not by any *T*_1_ fitting applied centrally to the submitted sets of *T*_1_ recovery images) were carried out using a bespoke MATLAB pipeline (The MathWorks Inc., Natick, Massachusetts, USA, R2012b) assembled in collaboration with the US National Institutes of Health. From the data, *T*_1_ for each of the nine tubes was measured in identically sized regions of interest (ROI) occupying the central 50% by area of each tube (accommodating ~ 40 independent pixels) and collated in a dedicated research electronic data capture instrument (REDCap [9, 10]).

### Methods part 3—Statistical analysis

Analysis was performed using R (version 3.0.1, R Foundation for Statistical Computing, Vienna, Austria). Descriptive data are expressed as mean ± standard deviation (SD) and standard error of the mean (SEM) as appropriate. Distribution of data were assessed on histograms and using the Shapiro-Wilk test.

#### Temperature sensitivity

Linear regression equations were used to relate temperature (predictor variable in degrees Celsius) and the response variable, phantom *T*_1_, by the formula: *T*1 = Intercept + (*β* ∗ [*Temperature* − 21 ° C]), with *β* representing the temperature correction, and 21 °C our arbitrarily chosen temperature for cross-center comparison.

#### Correlation with *rT*_1_ times

Correlations between estimated and *rT*_1_ times were derived using linear regression. Tests for significant inter-sequence and cross-vendor correlation differences (setting null value to 0.001) were conducted with alpha 0.01 and confidence level 0.95 [11].

#### *T*_1_ repeatability

After considering the normal values for native myocardial *T*_1_ reported in the published literature (e.g., in [12–16] as mean ± 1SD, though a 95% reference range is approximately ± 2SD), where 1 SD of the mean native myocardial *T*_1_ is generally ~ 20–30 ms at 1.5 T and ~ 50 ms at 3 T, we *arbitrarily* pre-defined as repeatable (and suitable for clinical/research use), *T*_1_ mapping approaches where the estimated variance of serial *T*_1_ data did not exceed ½ of the above in vivo 1SD. For *T*_1_ mapping at 1.5 T, this was ≤ 10 ms, i.e., CoV ≤ 1%; for *T*_1_ mapping at 3 T ≤ 25 ms, i.e., CoV ≤ 2%.

The CoV between serial repeat T1MES scans was calculated as the ratio of the SD to the mean. We appraised CoV as a compound measure of all causes of change in the estimated *T*_1_ of all nine tubes before and after temperature correction. We also appraised CoV after temperature correction separately for the four native and five post-GBCA tubes. Sequence-specific differences between the nine temperature-adjusted CoVs were calculated using paired *t* test with *P* value adjustment for multiple comparisons by the Bonferroni method (taking two-tailed *P* < 0.01 as significant).

#### Sources of *T*_1_ variation

Using temperature-adjusted *T*_1_ values of the “Medium” native myocardium tubes (tubes “F” and “M” respectively), we constructed linear mixed models to study the interplay of some known sources of *T*_1_ variation in multi-center phantom data. We did this separately for 1.5 T and 3 T phantom data. Considering temperature-adjusted *T*_1_ time as the response variable of interest, we examined the influence of phantom ID with and without the added effect of phantom age, as the combined fixed effect. With this, we then tested the following random effects:

i) Main effects and interactions of scanner vendor/scanner model (Siemens, Philips or General Electric [GE; General Electric Healthcare, Waukesha, Wisconsin, USA]; e.g., for Siemens: MAGNETOM Aera vs. Avanto vs. Espree, etc.);
ii) Main effects and interaction of sequence/scanner software version (considering all submitted variants of native modified Look-Locker inversion recovery [MOLLI] [17] sequences, shortened MOLLI [18] [ShMOLLI], native saturation-recovery single-shot acquisition [19] [SASHA], and saturation method using adaptive recovery times for cardiac *T*_1_ mapping [20] [SMART]; e.g., for Philips: R4.1.3SP2 vs. R5.1.7SP2 vs. R5.2.0SP2, etc.).

The response variable *T*_1_ fitted a normal probability distribution, so we estimated model parameters using maximum likelihood. ANOVA function using a type II Wald chi-square test evaluated the significance of fixed effects in the model. To compare models, Akaike and Bayesian information criteria (AIC, BIC) with the “smaller-is-better” criterion as well as chi-square values from inter-model ANOVA tests were used. The formulas used for model fitting and more definitions of the applied statistical tests are provided in Table 3.

#### Software upgrades

To explore whether software upgrades resulted in an abrupt “step” change in the temperature-adjusted *T*_1_ reads, we performed piece-wise linear regression to check for any segmented relationship between the covariates “scan day” and “tube *T*_1_” (considering tube “F” at 1.5 T and “M” at 3 T) [21]. For any broken-line relationship discovered, we defined slope parameters and break points where the linear relation/s changed and temporally correlated these with DICOM software metadata.

### Results part 1—Evaluation of the phantom

#### Structural integrity

Individual inspection of all the localizers submitted by sites with each phantom dataset revealed no visible gel rips or tears down any of the tubes. At baseline and at 2 years following batch manufacturing (Supplementary Movie 1), phantoms were free of air bubbles and susceptibility artifacts at both field strengths. High-resolution imaging showed no evidence of gel rips or tears down any of the tubes, and the gels were intact in the mid slice—the piloted location for serial mapping (Fig. 1, left panel). *T*_1_ maps collected through the midline of the phantom, using the specified T1MES scan setup, were free from off-resonance artifacts.

#### “Reference” *rT*_1_ and *rT*_2_ data

Phantom measurements (averaged across all tubes) collected at the Royal Brompton Hospital showed that temperature-corrected *rT*_1_ and *rT*_2_ values at 2 years were stable compared to baseline. At 1.5 T, the 2-year temperature-adjusted %*T*_1_ change was 1.95 ± 1.39% SD, 0.37% SEM. At 3 T, the 2-year temperature-adjusted %*T*_1_ change was 2.22 ± 1.44% SD, 0.25% SEM. Three tesla measurements at PTB showed a 2-year temperature-adjusted %*T*_1_ change of 0.80 ± 0.49%, SEM 0.16% (Supplementary Figure S1A). Although not the aim of this work, all reference *T*_2_ parameters remained stable with relative < 1% change at PTB (temperature-adjusted %*T*_2_ change over 2 years at 3 T = 1.65 ± 0.26% | 0.09%, Supplementary Figure S1B) and < 2% change over 2 years at Royal Brompton Hospital.

**Fig. 4:**
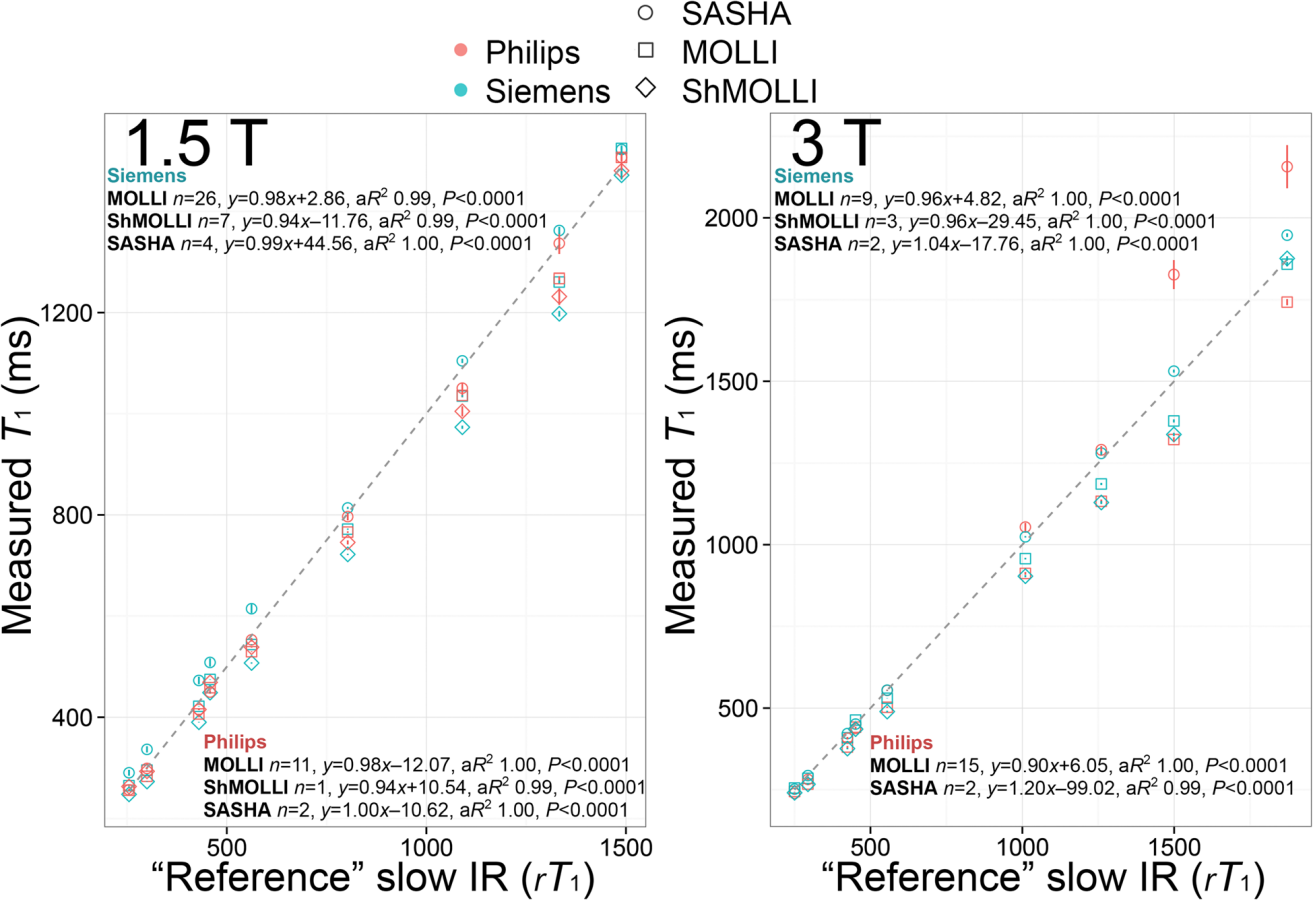
*T*_1_ times in the nine tubes for the three main sequence types (modified Look-Locker with inversion recovery (MOLLI), shortened MOLLI (ShMOLLI), saturation recovery single-shot acquisition (SASHA)) at 1.5 T (left) and 3 T (right) split according to vendor (Siemens, Philips). At 3 T, the contributed Philips MOLLI 5b(1b)1b(1b)1b was missing the iterative/data dropping steps in map creation as per Siemens ShMOLLI, so these data are not shown. Measured *T*_1_ times by the 3 sequences (mean of multiple centers, no temperature correction) are represented by symbols. The line of identity, i.e., “reference” *rT*_1_ times by slow inversion recovery is represented as a discontinuous gray line. For each sequence, correlations between absolute measurements of *T*_1_ and *rT*_1_ times are shown. Correlations for the much sparser GE data (MOLLI, ShMOLLI, SMART each *n* = 1) are not reported. Vertical error bars within each shape represent standard error of the mean. a*R*, adjusted *R*^2^; GE, General Electric Healthcare

Sequences from Siemens and Philips at 1.5 T collected at Royal Brompton Hospital showed strong correlation with *rT*_1_ times with small offsets, as shown in Fig. 4 (left panel). There were no statistically significant differences between the *rT*_1_ correlations for a given sequence when comparing Siemens to Philips platforms (Supplementary Table 1, panel C) or when individual sequences were compared on Philips (Supplementary Table S1, panel B). However, on Siemens, *rT*_1_ correlations for SASHA were significantly stronger than for both MOLLI and ShMOLLI (Supplementary Table S1, panel A).

Sequences from Siemens and Philips at 3 T showed strong correlation with *rT*_1_ times but with visibly imperfect absolute measurement of *T*_1_ across tubes of longer *T*_1_ times as shown in Fig. 4 (right panel). The correlations between absolute measurements of *T*_1_ and *rT*_1_ were similar for MOLLI and SASHA on Philips (Supplementary Table S2, panel B). On Siemens, the correlation between absolute measurements of *T*_1_ and *rT*_1_ was significantly stronger for SASHA when compared to MOLLI and ShMOLLI (Supplementary Table S2, panel A). The correlation between absolute measurements of *T*_1_ and *rT*_1_ for SASHA was significantly stronger on Siemens compared to Philips (Supplementary Table S2, panel C). Comparison of errors at 3 T relative to 1.5 T showed no significant difference (average SEM 5.49 vs. 4.04 respectively, *P* = 0.428).

#### Temperature sensitivity

Temperature experiments at both NIST and PTB using spin echo, long TR, and sequences (Fig. 2i) consistently showed that short-*T*_1_ tubes (modeling post-GBCA myocardium and blood) were more stable with temperature than very long-*T*_1_ tubes (native blood), where *T*_1_ increased more significantly with temperature. *T*_1_ increased by ~ 11 ms/°C for a typical MOLLI 5s(3s)3s 1.5 T dataset in tube “B” (normal native blood). Conversely, *T*_1_ decreased by ~ 1.2 ms/°C in tube “G” (short post-GBCA myocardium). Temperature corrections for each of the nine tubes at both field strengths from the pooled multi-center analysis are presented separately (Supplementary Table S3) from temperature sensitivity experiments of NIST and PTB due to the latter’s use of bespoke standard operating procedures.

#### *B*_0_ and *B*_1_ uniformity

**Fig. 5:**
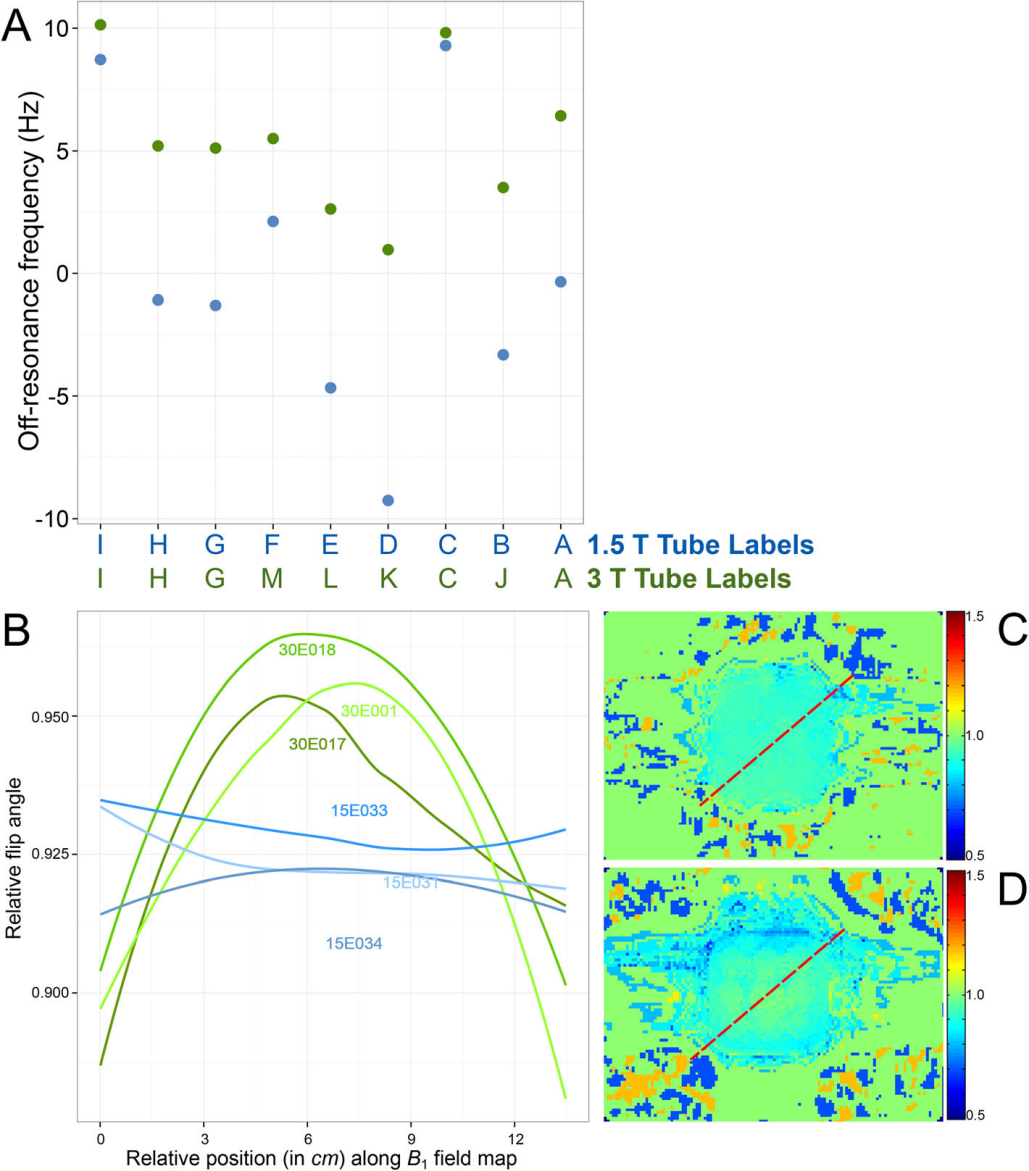
**a***B*_*0*_ field homogeneity at 2 years post manufacture across the nine phantom compartments as a measure of off-resonance in hertz at 1.5 T (blue, averaged for three phantoms) and 3 T (green, averaged for another three phantoms). These are extremely small shifts in frequency (e.g., 10 Hz = 0.08 ppm at 3 T) and should not be regarded as significantly different between the tube compartments. **b** Diagonal profiles of the *B*_1_ field at 2 years post manufacture as per red discontinuous lines (right panels) in six phantoms: three at 1.5 T scanned on 1.5 T MAGNETOM Avanto (Siemens Healthineers; software syngo MR B17A); example field map (**c**) and three at 3 T scanned on 3 T MAGNETOM Skyra (Siemens Healthineers; software syngo MR D13C); example field map (**d**)

*B*_*0*_ uniformity at 2 years was delivered to within ± 9 Hz at 1.5 T and ± 10 Hz at 3 T (Fig. 5a–b). At 2 years after manufacture, the *B*_1_ field distortion caused by the phantom, continued to be adequately flattened to within 10% of the *B*_1_ at the center of the phantom, at both 1.5 T and 3 T (Fig. 5c–d).

### Results part 2—Multi-center phantom testing

Thirty-four of the magnet systems appraised were 1.5 T and 22 were 3 T (see Supplementary Data File and Supplementary Table S4) with manufacturers being 35 Siemens, 18 Philips, and 3 GE. Scan frequencies are reported in the Supplementary Data File. The planned program was 1 year, but 24 centers voluntarily extended, resulting in mean longitudinal data of 358 days, and the longest 1161 days.

Quality assurance of the contributed DICOM image files identified significant protocol deviations or sequence reconstruction failures in nine submissions, the majority of which took place at the start of the study (examples in Supplementary Fig. S2). These were excluded with subsequent advice sent to centers permitting later inclusion in most cases, except where a tube artifact affecting > 25% of a single center’s series led to exclusion of that tube from pooled analysis (see Supplementary Data File).

Fifty-two magnets non-exclusively contributed MOLLI datasets, 16 ShMOLLI, and 12 SASHA, and 2 magnets contributed SMART *T*_1_ maps. Center-, session-, and sequence-specific *T*_1_ mapping contributions are detailed in Supplementary Data File. During the project, there were three software upgrades at centers: two Siemens and one GE (details in Supplementary Fig. S4).

#### *T*_1_ repeatability

For serial multi-center data, temperature-unadjusted CoV per tube, per phantom, per sequence, and per magnet are detailed in the Supplementary Data File. Temperature-adjusted CoV for the various native sequences together with inter-sequence and cross-platform differences, at 1.5 T and 3 T, are summarized in Tables 1 and 2 respectively and data for post-GBCA in Supplementary Tables S5 and S6. Results of native and post-GBCA sequence/scanner software version repeatability are provided in Tables 1 and 2 and in Supplementary Tables S5 and S6 respectively.

**Table 1:** Temperature-adjusted (normalized to 21 °C) native *T*_1_ and CoV (%) at 1.5 T summarized by vendor and sequence

**Summary table of*x̅*CoV of*T***_**1**_**(%) across 4 tubes**			**Order of tube IDs follows their orientation in the scanned bottle**			
Siemens	MOLLI 5s(3s)3s [448B]	**0.27**				
Philips	MOLLI 3s(3s)5s	**0.54**				
Siemens	SASHA	**0.56**				
Siemens	ShMOLLI 5b(1b)1b(1b)1b [1041B]	**0.64**				
Philips	SASHA	**0.92**				
Philips	ShMOLLI 5b(1b)1b(1b)1b^c^	**1.04**				
General Electric	MOLLI 5b(1b)1b(1b)1b^d^	**1.28**				
General Electric	SMART	**3.00**				
**Platform**	**Sequence [prototype, #]**		***x̅*’ CoV of*T***_**1**_**(%), global*x̅*± SD of*T***_**1**_**according to native tube ID** [tube *rT*_1_ by slow IR]			
			**F** [1090 ms]	**E** [1333 ms]	**D** [803 ms]	**B** [1489 ms]
**Siemens**	MOLLI MyoMaps product 5s(3s)3s [5]		*1.07*, *1041 ± 23*	*1.38*, *1273 ± 35*	0.85, 771 ± 6	*1.45*, *1538 ± 46*
	MOLLI 3b(3s)3b(3s)5b [448, 3]		0.87, 1019 ± 11	*1.08*, *1225 ± 17*	0.65, 765 ± 6	*1.19*, *1462 ± 21*
	MOLLI 5b(3s)3b [448, 3]		0.18, 1005 ± 13	0.53, 1217 ± 24	0.26, 762 ± 6	0.74, 1464 ± 23
	MOLLI 5b(3s)3b [448B, 2]		0.65, 1052 ± 7	0.82, 1289 ± 10	0.61, 784 ± 4	1.00, 1555 ± 16
	MOLLI 5b(3s)3b [780, 2]		0.70, 1048 ± 14	*1.18*, *1276 ± 20*	0.61, 776 ± 6 sic*.*	*1.15*, *1536 ± 24*
	MOLLI 5b(3s)3b [780B, 4]		0.76, 1043 ± 9	0.99, 1279 ± 17	0.67, 776 ± 6	*1.45*, *1550 ± 37*
	MOLLI 5s(3s)3s [448, 2]		0.89, 1035 ± 10	*1.51*, *1261 ± 25*	*1.04*, *770 ± 9*	*1.44*, *1538 ± 24*
	MOLLI 5s(3s)3s [448B, 1]^a^		0.14, 1051 ± 1	0.38, 1282 ± 5	0.17, 783 ± 1	0.38, 1553 ± 6
	MOLLI 5s(3s)3s [780B, 2]		0.81, 1043 ± 15	0.69, 1277 ± 15	0.42, 771 ± 6	0.90, 1546 ± 25
	MOLLI 5s(3s)3s [1041, 1]		*1.11*, *1045 ± 12*	0.69, 1285 ± 9	0.81, 772 ± 6	0.96, 1560 ± 15
	MOLLI 5s(3s)3s [1041B, 1]		*1.27*, *1033 ± 13*	*1.80*, *1232 ± 22*	0.66, 771 ± 5	*1.47*, *1494 ± 22*
	ShMOLLI 5b(1b)1b(1b)1b [448, 1]		*1.01*, *989 ± 10*	*1.39*, *1223 ± 17*	0.60, 729 ± 4	*1.50*, *1510 ± 23*
	ShMOLLI 5b(1b)1b(1b)1b [448C, 1]		0.61, 983 ± 6	0.64, 1218 ± 8	0.63, 727 ± 5	*1.57*, *1507 ± 22*
	ShMOLLI 5b(1b)1b(1b)1b [780B, 3]		*2.16*, *945 ± 34*	*3.29*, *1147 ± 62*	0.98, 711 ± 11	*3.70*, *1392 ± 85*
	ShMOLLI 5b(1b)1b(1b)1b [1048, 1]		0.70, 972 ± 7	0.71, 1208 ± 9	0.68, 715 ± 5	*1.01*, *1489 ± 15*
	ShMOLLI 5b(1b)1b(1b)1b [1041B, 1]^a^		0.59, 978 ± 6	0.67, 1201 ± 8	0.43, 721 ± 3	0.86, 1501 ± 13
	SASHA [4]		0.39, 1104 ± 17	0.65, 1362 ± 27	0.47, 814 ± 8	0.73, 1522 ± 30
**Philips**	MOLLI CardiacQuant product 5s(3s)3s [2]		0.53, 1025 ± 13	0.86, 1265 ± 19	0.50, 760 ± 7	*1.04*, *1516 ± 28*
	MOLLI 3b(3s)3b(3s)5b [2]		0.47, 1006 ± 17	0.94, 1247 ± 39	0.46, 751 ± 6	*1.25*, *1503 ± 46*
	MOLLI 3s(3s)3s(3s)5s [1]		0.94, 1026 ± 10	*1.66*, *1251 ± 21*	0.69, 765 ± 5	*2.15*, *1450 ± 31*
	MOLLI 3s(3s)5s [1]^a^		0.20, 936 ± 2	*1.11*, *1100 ± 12*	0.27, 722 ± 2	0.59, 1323 ± 8
	MOLLI 5b(3s)3b(3s)2b [1]		*6.78*, *1168 ± 79*	*6.32*, *1374 ± 87*	*3.04*, *816 ± 25*	*6.53*, *1665 ± 109*
	MOLLI 5b(3s)3b [1]		*1.18*, *1027 ± 12*	*1.48*, *1265 ± 19*	0.67, 758 ± 5	*1.16*, *1522 ± 18*
	MOLLI 5 s(3s)3s [3]		0.89, 1012 ± 17	*1.35*, *1233 ± 31*	*1.20*, *756 ± 9*	*1.76*, *1456 ± 58*
	ShMOLLI 5b(1b)1b(1b)1b^c^ [1]		0.89, 1027 ± 9	*1.37*, *1270 ± 17*	0.50, 753 ± 4	*1.41*, *1522 ± 21*
	SASHA [2]		*1.02*, *1057 ± 44*	0.89, 1336 ± 84	0.87, 798 ± 30	0.89, 1505 ± 33
**General Electric**	MOLLI 5b(3s)5b [1]		*11.18*, *653 ± 73*	*7.69*, *853 ± 66*	*8.45*, *591 ± 50*	*6.68*, *1319 ± 88*
	MOLLI 5b(1b)1b(1b)1b^d^ [1]^a^		*1.40*, *575 ± 8*	*1.76*, *642 ± 11*	0.78, 543 ± 4	*1.19*, *1002 ± 12*
	SMART [1]		*2.05*, *1063 ± 22*	*4.76*, *1273 ± 61*	0.95, 798 ± 8	*4.22*, *1430 ± 60*

The term sequence refers to either MOLLI, ShMOLLI, SASHA, or SMART

MOLLI/ShMOLLI protocol nosology has the number of inversions per experiment as the total count of numbers outside brackets, image cycles are outside brackets, pause cycles are within brackets, and cycle lengths defined in terms of either heart beats (b) or seconds (s)

*CoV* coefficient of variation, *ID* identity code, *GBCA* gadolinium-based contrast agent, *GE* General Electric, *MOLLI* modified Look-Locker inversion recovery, *rT*_*1*_ “reference” slow inversion recovery *T*_1_, *SASHA* saturation-recovery single-shot acquisition, *SD* standard deviation, *ShMOLLI* shortened MOLLI, *SMART* saturation method using adaptive recovery times for cardiac *T*_1_ mapping, *T* Tesla

^a^Denotes the *T*_1_ mapping sequence|software combination with lowest overall CoV% for a given vendor where multiples exist. *P* values for differences in CoV between sequences/vendors are reported for this highly repeatable sequence where multiples exist. Less favorable CoVs (> 1%, see the “*T*_*1*_ repeatability” section) are in italics. Post-GBCA tubes are not shown here as their data are reported separately in relation to post-GBCA sequences in Supplementary Table 5

^b^Denotes the number of different magnets submitting that particular sequence from which the average CoVs were derived

^c^Using iterative/data dropping steps in map creation as per ShMOLLI

^d^In the absence of iterative/data dropping steps in map creation as per ShMOLLI

*x̅* = average CoV across the 4 native tubes for a given sequence

*x̅*’ = where more than one sequence type was submitted, individual CoVs were then averaged to derive *x̅*’ CoV; while for single sequence submissions *x̅’* CoV is from the global mean *T*_1_ ± SD for that one sequence

**Table 2:** Temperature-adjusted (normalized to 21 °C) native *T*_1_ and CoV (%) at 3 T summarized by vendor and sequence

**Summary table of*x̅*CoV of*T***_**1**_**(%) across 4 tubes**			**Order of tube IDs follows their orientation in the scanned bottle**			
**Philips**	MOLLI 3b(3s)5b	0.33				
**Siemens**	ShMOLLI 5b(1b)1b(1b)1b [780C]	0.69				
**Siemens**	MOLLI 5b(3s)3b [448B]	0.95				
**Siemens**	SASHA	1.29				
**Philips**	SASHA	3.76				
**GE**	SMART	3.76 sic*.*				
**Platform**	**Sequence [prototype, #]**		***x̅*’ CoV of*T***_**1**_**(%), global*x̅*± SD of*T***_**1**_**according to native tube ID** [tube *rT*_1_ by slow IR]			
			**M** [1260 ms]	**L** [1499 ms]	**K** [1010 ms]	**J** [1872 ms]
**Siemens**	MOLLI MyoMaps product 5s(3s)3s [4]		1.28, 1182 ± 20	1.61, 1367 ± 29	1.02, 955 ± 13	*2.42*, *1831 ± 62*
	MOLLI 3b(3s)3b(3s)5b [448, 1]		1.16, 1092 ± 13	1.92, 1252 ± 24	0.56, 889 ± 5	1.58, 1724 ± 27
	MOLLI 5b(3s)3b [448B, 1]^a^		0.36, 1233 ± 4	0.78, 1449 ± 11	0.76, 986 ± 7	0.86, 2012 ± 17
	MOLLI 5b(3s)3b [780B, 2]		0.99, 1204 ± 14	1.13, 1410 ± 18	0.74, 970 ± 9	1.50, 1912 ± 28
	MOLLI 5s(3s)3s [780B, 1]		1.46, 1222 ± 18	*2.36*, *1435 ± 34*	1.76, 980 ± 17	*3.04*, *1916 ± 58*
	ShMOLLI 5b(1b)1b(1b)1b [780B, 2]		1.17, 1155 ± 27	1.62, 1361 ± 35	0.80, 922 ± 17	*2.45*, *1882 ± 41*
	ShMOLLI 5b(1b)1b(1b)1b [780C, 1]^a^		0.78, 1109 ± 9	0.89, 1322 ± 12	0.64, 888 ± 6	1.51, 1892 ± 29
	SASHA [2]		*2.04*, *1284 ± 30*	1.15, 1534 ± 27	0.70, 1025 ± 13	1.27, 1949 ± 35
**Philips**	MOLLI 3b(3s)3b(3s)5b [4]		1.64, 1147 ± 52	*2.42*, *1343 ± 80*	1.05, 923 ± 29	*3.90*, *1785 ± 115*
	MOLLI 3b(3s)5b [1]^a^		0.01, 1055 ± 1	0.49, 1217 ± 6	0.05, 862 ± 1	0.78, 1710 ± 13
	MOLLI 3s(3s)5s [1]		*5.41*, *1175 ± 64*	*3.72*, *1392 ± 52*	*7.13*, *937 ± 67*	*3.56*, *1833 ± 65*
	MOLLI 5b(3s)3b [3]		*4.18*, *1098 ± 48*	*5.44*, *1286 ± 73*	*4.37*, *868 ± 42*	*6.33*, *1784 ± 106*
	MOLLI 5s(3s)3s [6]		*2.40*, *1131 ± 70*	*2.41*, *1320 ± 108*	*2.60*, *906 ± 34*	*2.71*, *1727 ± 131*
	MOLLI 5b(1b)1b(1b)1b^c^ [2]		*2.98*, *948 ± 97*	*4.53*, *1082 ± 137*	0.96, 794 ± 60	*6.29*, *1286 ± 282*
	SASHA [2]		*3.11*, *1290 ± 46*	*3.40*, *1826 ± 171*	*2.11*, *1054 ± 38*	*6.41*, *2157 ± 267*
**General Electric**	SMART [1]		*2.85*, *1150 ± 33*	*4.13*, *1227 ± 51*	*2.04*, *973 ± 20*	*6.03*, *1419 ± 86*

Less favorable CoVs (> 2%, see the “*T*_*1*_ repeatability” section) are in italics. Post-GBCA tubes are not shown here as their data are reported separately in relation to post-GBCA sequences in Supplementary Table 6

sic = text is quoted exactly as it stands in the original, i.e., this is not a typo. Abbreviations as in Table 1

^a^Denotes the *T*_1_ mapping sequence|software combination with lowest overall CoV% for a given vendor where multiples exist

^b^Denotes the number of different magnets submitting that particular sequence from which the average CoVs were derived

^c^In the absence of iterative/data dropping steps in map creation as per ShMOLLI

*x̅* = average CoV across the 4 native tubes for a given sequence

*x̅*’ = where more than one sequence type was submitted, individual CoVs were then averaged to derive *x̅’* CoV; while for single sequence submissions *x̅’* CoV is from the global mean *T*_1_ ± SD for that one sequence

Based on these temperature-adjusted data, mean (*x̅*) CoV were generally higher (implying poorer repeatability) at 3 T than at 1.5 T and for the much sparser GE data over Siemens/Philips. Over 1 year, many 1.5 T and 3 T sequences/magnets were repeatable with *x̅* CoV < 1% and < 2% respectively. For sequences optimized for native *T*_1_ mapping applied to T1MES native tubes, we observed excellent repeatability for several sequences, for example, Siemens MOLLI 5s(3s)3s 448B (*x̅* CoV = 0.27%) and Philips MOLLI 3s(3s)5s (*x̅* CoV 0.54%) at 1.5 T (Table 1) and Philips MOLLI 3b(3s)5b (*x̅* CoV 0.33%) and Siemens ShMOLLI (*x̅* CoV 0.69%) at 3 T (Table 2).

Among sequences optimized for post-GBCA *T*_1_ mapping applied to T1MES post-GBCA tubes, excellent repeatability was observed for several sequences, for example, Siemens ShMOLLI 1041B (*x̅* CoV 0.21%) and Siemens MOLLI 4s(1s)3s(1s)2s 448B (*x̅* CoV 0.26%) at 1.5 T (Supplementary Table S5) and Siemens MOLLI 4b(1s)3b(1s)2b 448B (*x̅* CoV 0.10%) and Siemens ShMOLLI (*x̅* CoV 0.28%) at 3 T (Supplementary Table S5).

#### Sources of *T*_1_ variation

Linear mixed models which excluded centers that had experienced a software change during the time of data collection (Table 3 and Supplementary Tables S7 and S8) indicated that temperature-adjusted *T*_1_ differs significantly between sequences and software versions at both field strengths (*P* < 0.001 all) and between magnet models at 1.5 T (*P* = 0.011). Notably, phantom age had no significant effect on *T*_1_ (model A2).

**Table 3:** Linear mixed models for 1.5 T and 3 T multi-center temperature-adjusted *T*_1_ mapping data (normalized to 21 °C, considering the “Medium” native myocardium tubes “F” and “M” respectively). The best model is “A5” at 1.5 T and “A3” at 3 T

	Model fitting formulas		AIC	BIC	Log likelihood	***χ***^**2**^	***P*** value	Best model
**1.5 T model**	A1: *T*_1_ ~ sequence + (1|ID)		5850.3	5920.8	− 2909.1	“ref”	“ref”	“ref”
	A2: *T*_1_ ~ sequence + (1|ID/age) [worse fit with age]		5852.3	5927.2	− 2909.1	0.0	1.000	–
	A3: *T*_1_ ~ sequence × software + (1|ID)		5725.4	5883.9	− 2826.7	164.9	**< 0.0001**	–
	A4: *T*_1_ ~ sequence × software + vendor + (1|ID) [vendor non-contributory to fit]		5725.4	5883.9	− 2826.7	0.0	1.000	–
	A5: *T*_1_ ~ sequence × software + model + (1|ID)		5723.4	5890.8	− 2823.7	5.9	0.051	**A5**
	A6: *T*_1_ ~ sequence × software + vendor × model + (1|ID) [vendor non-contributory to fit]		5723.4	5890.8	− 2823.7	0.0	1.000	–
	**Final model (A5) parameters**		**Variance**	**SD**	***χ***^**2**^	***P*value**		
	**Random effects**	ID	2547.2	50.47	“ref”	“ref”		
	**Fixed effects**	Sequence (***β*** range *−* 32.3 to 455.6^a^*)*	/	/	1101.0	**< 0.0001**		
		Software (***β*** range 385.1 to 522.3^a^*)*	/	/	63.8	**< 0.0001**		
		Model (***β*** range − 161.8 to 16.0^a^*)*	/	/	11.2	**0.011**		
		Sequence: Software (***β*** range − 161.8 to − 6.9^a^*)*	/	/	149.6	**< 0.0001**		
**3 T model**	A1: *T*_1_ ~ sequence + (1|ID)		4390.4	4447.0	− 2181.2	“ref”	“ref”	“ref”
	A2: *T*_1_ ~ sequence + (1|ID/age) [worse fit with age]		4392.4	4453.1	− 2181.2	0.0	1.000	–
	A3: *T*_1_ ~ sequence × software + (1|ID)		4238.3	4339.4	− 2094.2	174.1	**< 0.0001**	**A3**
	A4: *T*_1_ ~ sequence × software + vendor + (1|ID) [vendor non-contributory to fit]		4238.3	4339.4	− 2094.2	0.0	1.000	–
	A5: *T*_1_ ~ sequence × software + model + (1|ID)		4239.2	4348.4	− 2092.6	3.1	0.212	–
	A6: *T*_1_ ~ sequence × software + vendor × model + (1|ID) [vendor non-contributory to fit]		4239.2	4348.4	− 2092.6	0.0	1.000	–
	**Final model (A3) parameters**		**Variance**	**SD**	**χ**^**2**^	***P*-value**		
	**Random effects**	ID	372.4	19.3	“ref”	“ref”		
	**Fixed effects**	Sequence (***β*** range − 114.5 to 38.5^a^*)*	/	/	732.8	**< 0.0001**		
		Software (***β*** range − 203.0 to 32.7^a^*)*	/	/	46.0	**< 0.0001**		
		Sequence: Software (***β*** range − 48.8 to 267.3^a^*)*	/	/	189.0	**< 0.0001**		

The symbol (1|ID) in the model formulas refers to the random effect of individual phantoms (by identity number). At 1.5 T models, A5 and A6 have equal AIC/BIC but given the lack of statistically significant *χ*^2^ improvement from A5 to A6 (*P* = 1.0); A5 is considered the most parsimonious model

*Age* refers to phantom age at scanning since date of manufacture, *AIC* Akaike information criterion, *BIC* Bayesian information criterion, *χ2* chi-square, *ref* reference, *SD* standard deviation

^a^Complete list of *β* coefficients for model A5 at 1.5 T and A3 at 3 T are provided in Supplementary Tables 7 and 8 respectively

#### Software upgrades

The two software upgrades on Siemens both occurred towards the end of longitudinal data submissions (Supplementary Fig. S4A, 15E031; 4B, 30E017), so the paucity of data points post-upgrade events precluded statistical testing for significant *T*_1_ shifts. For the software upgrade on GE, however, differences between pre- and post-linear regression slopes (Supplementary Fig. S4C, 30E012) indicate a marginally significant *T*_1_ shift event (*P* = 0.024).

## Discussion

To our knowledge, this is the first multi-center study using a wide range of *T*_1_ mapping methods to study the interplay of some of the known sources of measured *T*_1_ variation. The basis of this work is the T1MES phantom, and a major aim of this work was to evaluate how this performed in use at multiple centers. The phantom appears sufficiently robust for *T*_1_ mapping quality assurance purposes. Based on our data, estimated phantom *T*_1_ values may show substantial variation between different *T*_1_ mapping sequences, scanner software versions, and potentially also scanner models, and temperature correction, at least for native *T*_1_ tubes, is necessary to achieve the desired repeatability. Inspection of the Supplementary Table also suggests that there are occasional performance variations producing outliers even within a given *T*_1_ mapping sequence prototype/software combination across different magnets. In spite of this variation, however, several specific combinations of field strength, sequence, and scanner generally exhibit excellent repeatability. Given the choices, the CMR community may prefer to standardize and use combinations with high repeatability for future clinical and research use, e.g., CoV < 1% at 1.5 T and < 2% at 3 T, while seeking to optimize less repeatable combinations. Less data were available for GE in T1MES, so the observed variability requires verification in a larger more representative sample.

Agreement with “reference” slow scanning, *rT*_1_ times was slightly greater for SASHA compared to MOLLI or ShMOLLI, which both slightly underestimated *T*_1_, mainly from the known *T*_2_-related underestimation which is larger when measuring the myocardium [13–15], (Fig. 4, Supplementary Tables S1 and S2) and not due to magnetization transfer, which is negligible in these agar phantoms [22].

The phantom data presented here suggest that the “*T*_1_ standard” framework remains possible, but the wide variety of different sequence options, vendors, and field strengths distributed the data over too many categories for reliable modeling, unlike the “locked-down” approach mentioned below which does have that strong advantage. Further work will explore the transferability of clinical measurement based on in vitro phantom calibration.

T1MES is but one of a number of phantom objects that have been used or proposed to support *T*_1_ mapping quality assurance (elaborated in Table 4). Some alternatives that target native and post-GBCA myocardial and blood relaxation times in support of *T*_1_ mapping work are (1) Brompton phantom by Vassiliou et al. [23], (2) Hypertrophic Cardiomyopathy Registry [24] phantom (HCMR) by Piechnik et al., and (3) International Society for Magnetic Resonance in Medicine (ISMRM)/NIST MR imaging phantom [25].

**Table 4:** Comparison of recently reported phantoms for cardiac *T*_1_ mapping quality assurance

	Dedicated ***T***_**1**_ device			Combined ***T***_**1**_/***T***_**2**_ device
**Phantom**	HCMR (cardiac specific)	Brompton (cardiac specific)	T1MES (cardiac specific)	ISMRM/NIST system phantom (not cardiac specific)
**Field strength specificity**	☒	☒	☑	☒
**Tube ingredients**	NiCl_2_-doped agar + carrageenan	NiCl_2_-doped agar	NiCl_2_-doped agar	NiCl_2_-doped water, MnCl_2_-doped agar
**Structure**	9 *T*_1_/*T*_2_ tubes in an amber PVC sealed jar that is ~ 25% smaller in total volume compared to T1MES.	Air-filled box containing 4 duplicate *T*_1_/*T*_2_ glued glass tubes (total 8) requires cylindrical MRI test bottles on either side for *B*_1_ field/reference frequency calibration.	9 plastic *T*_1_/*T*_2_ tubes in an amber PVC sealed jar with inter-tube gaps packed with a carefully specified agarose/ HDPE plastic macrobead/NiCl_2_-doped fill that flattened the *B*_1_ field and provided sufficient *B*_0_ homogeneity to obviate the need for side phantoms.	A layer of 14 *T*_1_ spheres and a separate layer of 14 *T*_2_ spheres so no unified *T*_1_/*T*_2_ compartment representing the relaxation parameters of the human heart.
**Cardiac T**_**1**_**/T**_**2**_**coverage**	Health and disease, 9 biologies but limited *T*_2_ coverage (57 and 75 ms).	Health only, 4 biologies: (1) native myocardium, (2) native blood, (3) post-GBCA myocardium, and (4) post-GBCA blood.	Health and disease, 9 biologies and broad *T*_2_ coverage (1.5 T, 44, 48, 50, 155, 189, and 243 ms). Also see Supplementary Table 3.	Health and disease, but of the 14 *T*_1_ spheres (ranging from 21 to 2038 ms), half are not useful to cardiac *T*_1_ mapping as *T*_1_ times are too short for either native or post-GBCA myocardium/blood. Half the *T*_2_ spheres (ranging from 11 to 581 ms) are also anti-physiological for cardiac mapping. Also, the *T*_1_/*T*_2_ ratio is not representative of cardiac tissue.
**MT coverage**	–	–	–	–
**Regulatory clearance**	–	–	FDA, CE-mark	–
**Developers**	Piechnik et al.	Vassiliou et al.	Captur et al.	NIST/ISMRM

*CE* Conformitée-Europeen, *FDA* Food and Drug Administration, *HDPE* high-density polyethylene, *MT* magnetization transfer, *PVC* poly vinyl chloride

At 1 year, CoV for *T*_1_ tubes of the Vassiliou [26] phantom on a single Siemens scanner ranged from 1.0 to 3.6% using only the native MOLLI 5s(3s)3s sequence, compared to 0.27 to 3.0% in T1MES (for 1.5 T MOLLI 5s(3s)3s [448B] on Siemens to SMART on GE respectively). This within sequence precision heterogeneity, as already alluded to by Kellman et al. [27], linked to protocol modifications within MOLLI, may partly explain some of the higher CoV for specific MOLLI sequences compared to ShMOLLI where centers obligatorily scanned using a fixed 5b(1b)1b(1b)1b sequence with conditional fitting. As mentioned above for the dispersion of T1MES results, this “real-world” heterogeneity of *T*_1_ mapping sequences highlights that while *T*_1_ mapping research and innovation calls for multiple flexible prototypes, the resultant diversity poses a nontrivial challenge to multi-center standardization. Conversely, the more “locked-down” approach like that adopted for ShMOLLI, albeit less editable by external researchers, potentially facilitates standardization.

The design of phantoms for this purpose (although often seen as trivial) is challenging: to have a large sufficient ROI in each sample tube and a good number of sample tubes in the main jar of the phantom, the overall size of the phantom increases to the point that *B*_1_ non-uniformity at 3 T due to the dielectric permittivity of any water-based phantom becomes problematic. While of course *B*_1_ is non-uniform in vivo, one purpose of phantom tests is to eliminate uncertainty or at least enable controlled testing of factors such as *B*_1_ rather than introduce their own errors. Conversely, if the phantom is made very small, then the truly acquired pixel size of in vivo *T*_1_ mapping methods (which obviously must not be modified or adapted [28] for the phantom scans) becomes important compared to the sample tube inner diameters leading to questions over SNR [29] and the impact of Gibbs artifact, for example.

Another obstacle for relaxation time phantoms (on top of basic water *T*_1_ and *T*_2_ temperature and pressure variations, gel instabilities [30, 31], small leakages, dehydration, or ion migration effects over time) is the differing temperature dependence of each species of paramagnetic ion’s relaxivity (*r*_1_, *r*_2_) and furthermore frequency dispersion in all of these physical properties between 60 MHz and 120 MHz [32–36]. Efforts to use alternative chemistry have so far not provided the range of *T*_1_ and *T*_2_ needed for this application.

The results confirmed, as is widely known in the field, that the *T*_1_ measured by even nominally identical mapping sequences can differ significantly between software revisions. Scanner software upgrades are not uncommon in centers, and we encountered three such events among contributing partners. There was a measurable shift on at least one system, with potentially important implications for the field as alluded to in the 2017 consensus statement on multi-parametric mapping by the Society for Cardiovascular Magnetic Resonance (SCMR) [37]. Future work using phantoms at scale, across vendors and over time, would ideally continue monitoring center-specific data before and after potential shift events to more fully understand their impact on *T*_1_ allowing the community to set up mitigation approaches.

Clearly, sources of *T*_1_ variance and drift over time and between centers are numerous. Some *T*_1_ variance is related to differences between sequences and magnet platforms: to the scanner itself (equipment drift or equipment shifts from updates of software, replacement of parts, routine service recalibrations, etc.), to the environment (temperature, pressure, humidity), and to the operator (phantom positioning inside scanner bore). In a phantom study, some *T*_1_ variance may additionally have been due to intra-phantom differences (liquid/agar status over time collectively contributing to phantom aging) and inter-phantom manufacturing variations. In T1MES, we mitigated potential inter-phantom manufacturing variations by exercising extreme caution in design and prototyping and by the strict batch manufacturing processes to medical device grade standards. Drifts in the system setup (long-term stability) would tend to be “adjusted” out by the shim and center frequency (CF) and transmitter *B*_1_ reference (TxREF) adjustments that could otherwise bias estimated *T*_1_. Any receiver coil or gain changes would most likely cancel out of *T*_1_ maps as constant across the series of input images to *T*_1_ derivation (appearing in A and B maps instead). It is considered unlikely that gradient performance would drift significantly, and any significant sudden changes in that are also unlikely even when the eddy-current compensation is adjusted on routine service visits. However, we have shown how unforeseen software changes could potentially cause “step-wise” changes in the estimated *T*_1_ records. To directly model the interplay between all these potential sources of T_1_ variation in longitudinal phantom studies would be a daunting task, and we considered only a subset of these sources in our analyses.

Though *B*_0_ and *B*_1_ homogeneity tests in T1MES (Fig. 5) were stable, we did measure slightly greater field inhomogeneity at the phantom edge and corners compared to the core (phantom corners and edges are the zones which our T1MES data flagged up as areas of least *B*_0_/*B*_1_ homogeneity). The tubes with the highest *T*_1_ times are the tubes most susceptible to such field inhomogeneities, and this suggests that a future iteration of T1MES could be further optimized by rearrangement to position such tubes in the most uniform regions.

The results of this study are relevant to clinicians or researchers choosing sequences for *T*_1_ mapping, and we highlight 3 take home messages as follows:

i. Sequence/software combinations in Tables 1 and 2, with a CoV ≤ 1% and 2% respectively, are sufficiently repeatable for clinical/research use (see rationale in the “Methods part 3—Statistical analysis” and “*T*_*1*_ repeatability” sections). For the native myocardium T1MES tube at 1.5 T (reference T_1_ ~ 1037 ms by MOLLI), a ≤ 1% CoV is well within ± 1SD of normal values for *T*_1_, meaning that small-magnitude biological changes (e.g., diffuse myocardial fibrosis), will be resolvable with high precision [37]. By contrast, CoVs of 3 or 6% at 1.5 T (variance of 31 and 62 ms respectively) will undermine a center’s ability to resolve small-magnitude changes, and they may also impact the resolution of large-magnitude biological changes [37] (like amyloid, iron overload, Fabry disease, acute myocardial injury). Our data indicate that many of the investigated sequences for Siemens and Philips at 1.5 T exhibited CoVs ≤ 1%. At 3 T, MOLLI (Siemens and Philips), ShMOLLI (Siemens), and SASHA (Siemens) exhibited CoVs ≤ 2%. The absence of GE from this list is due to limited participation.
ii. For developers, the highlighting of sequence performance may encourage work to refine sequences in some cases.
iii. The prospect of using phantom calibration to remove the need for local reference ranges [37] is potentially becoming more tangible than before, yet more work is needed to deliver such calibration.

These data may facilitate the use of *T*_1_ mapping as a useful clinical biomarker. *T*_1_ standardization will also be important to facilitate clinical research. At the present time, there are 25 registered clinical research studies using *T*_1_ mapping (*clinicaltrials.gov* accessed January 2019).

### Limitations

Presented results are not intended to compare the SNR obtained in the phantom setup in different machines but are aimed at studying long-term stability. In spite of our outreach to advertise enrollment at study start, GE centers are under-represented. The scarcity of multi-center data for many of the specific *T*_1_ mapping sequence, software, and field-strength combinations has limited our ability to deliver a “*T*_1_ standard”. Eight centers enrolled, received a phantom, but then could not deliver the serial phantom scans, and four centers did not meet the 1 year minimum data submission requirement. Each center provided legitimate justifications for the missing data, and furthermore, we highlight that contributions to T1MES were entirely voluntary, with no center receiving remuneration for any staff or scanner time invested in the program.

In total, 3 magnets deviated from the prescribed nominal intervals in some of their initially submitted datasets. Phantom repeatability studies represent an in vitro experiment. Some sequences and scanners that performed well in vitro may exhibit greater variability in the face of biological tissue (magnetization transfer, flow, motion), patient characteristics (fast/slow heart rates, heart rate variability, breath-hold length, implanted metal devices), and scanning process (non-isocentering, scanning in multiple planes). The T1MES phantom does not capture magnetization transfer. The biomarker performance that was measured here accounts for *T*_1_ mapping in vitro. While this is obviously related to diagnostic performance in vivo*,* cardiac motion, etc., introduce yet another degree of freedom, and different hardware/software combinations might deal with this differently. In addition, CoV as a single metric may not be the best way to pick a pulse sequence for repeatability when the data is only derived from a static phantom. A very precise (reproducible) *T*_1_ sequence that requires long breatholds or is heart rate-variability-sensitive might perform very well with the T1MES phantom setup but perform poorly in patients with varying biology, e.g., that cannot hold their breath well or who are in atrial fibrillation. The phantom has no intracellular/extracellular component, so any researcher seeking to model “ECV” using pre- and post-GBCA *T*_1_ mapping values derived from T1MES (see example data in Supplementary Table S9) must bear this in mind.

The phantom design, ROI size, and coil setup instructions all aimed to make the fundamental image noise SNR an irrelevant factor in the CoV results. The artificially high and “clean” SNR of a phantom setup (if without phantom-related distortions, as we have shown in the “*B*_0_ and *B*_1_ uniformity” section) is clearly not directly an indicator of satisfactory in vivo performance. It contains many sequence-related factors that may have different consequences in vivo [29], and such SNR comparisons are *not* the aim of this work. Ideally, participating sites would have proved this by sending multiple *T*_1_ maps per session. However, contributions were already voluntary, and further demands on sites could not be supported. For the same reason, it was not feasible to demand long reference *T*_1_/*T*_2_ data for each participating site, and this was never part of the original enrollment commitment. As some indication of the typical short-term thermal noise random jitter, the raw (temperature-unadjusted) *T*_1_ values from some sites that did anyway submit several repeated *T*_1_ maps each session are plotted in Supplementary Fig. S3.

## Conclusion

The T1MES phantom developed to CE/FDA manufacturing standards for *T*_1_ mapping is a robust quality assurance device. *T*_1_ mapping can now be quality assured on a multi-center scale, fulfilling a key requirement for the use of *T*_1_ mapping in clinical decision-making or as a surrogate endpoint in drug trials. A good number of *T*_1_ measurement sequence/scanner model/vendor/field strength combinations are remarkably repeatable, but some combinations less so, with coefficients of variation exceeding 1–2% over 1 to 2 years. Given the alternatives, we recommend that combinations with poor repeatability are deprioritized for clinical and research use. In spite of the use of a large number of different sequence prototypes and products in this study, a number of agreements and encouraging results are reported. Phantom calibration of *T*_1_ mapping which obviates the need for local reference ranges, enabling the establishment of a “*T*_1_ standard” to facilitate multi-center *T*_1_ studies, comes closer, with further work required to address this.

## Supplementary information

**Additional file 1: Supplementary Movie 1.** High-resolution imaging of phantom 30E017 at baseline (October 2015, **Left panel**) and at two years post manufacturing (**Right panel**). (MP4 69,750 kb)

**Additional file 2:** Supplementary file containinglist of Supplementary Tables and Figures referenced in the main text.

**Additional file 3.** Supplementary data file reporting all the center-, session- and sequence-specific *T*_1_ mapping contributions to the T1MES program.

## Data Availability

Datasets collected for the current study are available from the corresponding author upon reasonable request.

## Abbreviations

3D: Three-dimensional
AIC: Akaike information criteria
BIC: Bayesian information criteria
CE: Conformité Européenne
CF: Center frequency
CMR: Cardiovascular magnetic resonance
CoV: Coefficient of variation
DICOM: Digital Imaging and COmmunications in Medicine
ECV: Extracellular volume
FA: Flip angle
FDA: Food and Drug Administration
GBCA: Gadolinium-based contrast agent
GE: General Electric Healthcare
HCMR: Hypertrophic Cardiomyopathy Registry
INR: International normalized ratio
IRSE: Inversion recovery spin echo
ISMRM: International Society for Magnetic Resonance in Medicine
MOLLI: Modified Look-Locker inversion recovery
NIST: National Institute of Standards and Technology
PTB: Physikalisch-Technische Bundesanstalt
QIBA: Quantitative Imaging Biomarkers Alliance
REDCap: Research electronic data capture
ROI: Region of interest
RSNA: Radiological Society of North America
*rT*_1_: “Reference” *T*_1_
*rT*_2_: “Reference” *T*_2_
SASHA: Saturation recovery single-shot acquisition
SCMR: Society for Cardiovascular Magnetic Resonance
SD: Standard deviation
ShMOLLI: Shortened MOLLI
SMART: Saturation method using adaptive recovery times for cardiac *T*_1_ mapping
SNR: Signal-to-noise ratio
T: Tesla
T1MES: *T*_1_ mapping and extracellular volume standardization
TE: Echo time
TR: Repetition time
TxREF: Transmitter *B*_1_ reference
