# Supplementary material for: T1 mapping performance and measurement repeatability: results from the multi-national T1 mapping standardization phantom program (T1MES)
Source: J Cardiovasc Magn Reson. 2020 May 7;22:31. doi: 10.1186/s12968-020-00613-3 (PMC7204222; doi:10.1186/s12968-020-00613-3)
Supplement: Supplementary file 2 — Additional file 2: Supplementary file containinglist of Supplementary Tables and Figures referenced in the main text. [file 12968_2020_613_MOESM2_ESM.docx]

TITLE

*T*_1_ Mapping Performance and Measurement Repeatability: Results from the Multi-National *T*_1_ Mapping Standardization Phantom Program (T1MES)

­AUTHORS: Gabriella Captur,^1,2,3^ Abhiyan Bhandari,^4^ Rüdiger Brühl,^5^ Bernd Ittermann,^5^ Kathryn E. Keenan,^6^ Ye Yang,^7^ Richard J. Eames,^8^ Giulia Benedetti,^9^ Camilla Torlasco,^10^ Lewis Ricketts,^4^ Redha Boubertakh,^11^ Nasri Fatih,^1,2^ John P. Greenwood,^12^ Leonie E.M. Paulis,^13^ Chris B. Lawton,^14^ Chiara Bucciarelli-Ducci,^14^ Hildo J Lamb,^15^ Richard Steeds,^16^ Steve W. Leung,^17^ Colin Berry,^18^ Sinitsyn Valentin,^19^ Andrew Flett,^20^ Charlotte de Lange,^21^ Francesco DeCobelli,^22^ Magalie Viallon,^23^ Pierre Croisille,^24^ David M. Higgins,^25^ Andreas Greiser,^26^ Wenjie Pang,^27^ Christian Hamilton-Craig,^28^ Wendy E Strugnell,^28^ Tom Dresselaers,^29^ Andrea Barison,^30^ Dana Dawson,^31^ Andrew J. Taylor,^32,33,34^ François-Pierre Mongeon,^35^ Sven Plein,^12^ Daniel Messroghli,^36,37^ Mouaz Al-Mallah,^38^ Stuart M. Grieve,^39^ Massimo Lombardi,^40^ Jihye Jang,^41^ Michael Salerno,^42^ Nish Chaturvedi,^2^ Peter Kellman,^43^ David A. Bluemke,^44^ Reza Nezafat,^41^ Peter Gatehouse,^45^ James C. Moon,^1,46^ on behalf of the T1MES Consortium

INSTITUTIONS

1. UCL Institute of Cardiovascular Science, University College London, Gower Street, London WC1E 6BT, UK

2. UCL MRC Unit for Lifelong Health and Ageing, University College London, 1-19 Torrington Place, London WC1E 7BH, UK

3. The Royal Free Hospital, Centre for Inherited Heart Muscle Conditions, Cardiology Department, Pond Street, Hampstead, London NW3 2QG, UK

4. UCL Medical School, Bloomsbury Campus, University College London, Gower Street, London WC1E 6BT, UK

5. Physikalisch-Technische Bundesanstalt (PTB), Abbestr. 2–12, D-10587 Berlin, Germany

6. National Institute of Standards and Technology (NIST), Boulder, MS 818.03, 325 Broadway, Boulder, CO, USA

7. Department of Cardiology, Sir Run Run Shaw Hospital, Zhejiang University, Hangzhou 310016, Zhejiang, PR China.

8. Department of Physics, Imperial College London, Prince Consort Rd, London SW7 2BB, UK

9. Department of Radiology, Guys and St Thomas NHS Foundation Trust, London, UK

10. University of Milan-Bicocca, Piazza dell’Ateneo Nuovo 1, 20100, Milan, Italy

11. Cardiovascular Biomedical Research Unit, Queen Mary University of London, London E1 4NS, UK

12. Multidisciplinary Cardiovascular Research Center & Division of Biomedical Imaging, Leeds Institute of Cardiovascular and Metabolic Medicine, University of Leeds, Leeds, UK

13. Department of Radiology & Nuclear Medicine, Maastricht University Medical Centre, PO Box 5800, 6202AZ Maastricht, The Netherlands

14. Bristol Heart Institute, National Institute of Health Research (NIHR) Biomedical Research Centre, University Hospitals Bristol NHS Foundation Trust and University of Bristol, Upper Maudlin St, Bristol, BS2 8HW, UK

15. Leiden University Medical Centre, Department of Radiology, Albinusdreef 2, 2333 ZA Leiden, The Netherlands

16. University Hospitals Birmingham NHS Foundation Trust, Edgbaston, Birmingham B15 2TH, UK

17. UK Albert B. Chandler Hospital - Pavilion G, Gill Heart & Vascular Institute, Lexington KY 40536, USA

18. Institute of Cardiovascular and Medical Sciences, RC309 Level C3, Bhf Gcrc, Glasgow, G12 8TA, Scotland, UK

19. Lomonosov Moscow State University, Department of Multidisciplinary Clinical Studies, Moscow, Russia

20. University Hospital Southampton Foundation Trust, Tremona Road, Southampton, Hampshire SO16 6YD, UK

21. Oslo University Hospital, Department of Radiology and Nuclear Medicine, Sognsvannsveien 20, 0372 Oslo, Norway

22. San Raffaele Hospital, Via Olgettina 60, 20132 Milan, Italy

23. University of Lyon, UJM-Saint-Etienne, INSA, CNRS UMR 5520, INSERM U1206, CREATIS, F-42023, Saint-Etienne, France

24. Department of Radiology, University Hospital Saint-Etienne, Saint-Etienne, France

25. Philips, Philips Centre, Unit 3, Guildford Business Park, Guildford, Surrey, GU2 8XG

26. SiemensHealthcare GmbH, Erlangen, Germany

27. Resonance Health, 278 Stirling Highway, Claremont, WA 6010, Australia

28. The Prince Charles Hospital, Griffith University and University of Queensland, Brisbane, Australia

29. Department of Radiology, Universitair Ziekenhuis Leuven, UZ Leuven, Belgium

30. Fondazione Toscana Gabriele Monasterio, Pisa, Italy

31. Polwarth Building School of Medicine and Dentistry University of Aberdeen, Foresterhill Aberdeen AB25 2ZD, Scotland, UK

32. Department of Cardiovascular Medicine, Alfred Hospital, Melbourne Australia

33. Baker Heart and Diabetes Institute, Melbourne Australia

34. Department of Medicine, Monash University, Melbourne Australia

35. Department of Medicine, Montreal Heart Institute and Université de Montréal, 5000 Bélanger Street, Montreal QC, Canada H1T 1C8

36. Department of Internal Medicine - Cardiology, Deutsches Herzzentrum Berlin

37. Department of Internal Medicine and Cardiology, Charité - Universitätsmedizin Berlin, Campus Virchow Klinikum

38. King Abdulaziz Cardiac Center (KACC) (Riyadh), National Guard Health Affairs, Kingdom of Saudi Arabia

39. The University of Sydney School of Medicine, NSW 2006, Australia

40. I.R.C.C.S., Policlinico San Donato, Piazza Edmondo Malan, 2, 20097 San Donato Milanese MI, Italy

41. Department of Medicine (Cardiovascular Division) Beth Israel Deaconess Medical Center, Harvard Medical School, Cardiology East Campus, Room E/SH455, 330 Brookline Ave, Boston, MA, 02215, USA

42. University of Virginia Health System, 1215 Lee St, PO Box 800158, Charlottesville VA 22908

43. National Heart, Lung, and Blood Institute, National Institutes of Health, Bethesda, MD 20892-1061, USA

44. Department of Radiology, University of Wisconsin School of Medicine and Public Health, Madison, WI 53792-3252, USA

45. CMRI Department, Royal Brompton Hospital, Sydney Street, London SW3 6NP, UK

46. Barts Heart Center. St Bartholomew’s Hospital, West Smithfield, London EC1A 7BE, UK

**Supplementary Table 1.** Differences between the correlations of the absolute measurement of *T­_1_* and “reference *T_1_*” (*rT_1_*) for a given sequence at 1.5T when compared to an alternative sequence on the same platform (**Panels A** and **B**) or to a similar sequence on a different platform (**Panel C**). *rT*_1_ times were obtained by slow inversion recovery at Royal Brompton Hospital. GE and Agilent not included (too few centers).

| **A** |  | **1.5T Siemens** | | |
| --- | --- | --- | --- | --- |
|  |  | **MOLLI**  a*R*^2^ = 0.9939 | **ShMOLLI**  a*R*^2^ = 0.9907 | **SASHA**  a*R*^2^ = 0.9991 |
| **1.5T Siemens** | **MOLLI**  a*R*^2^ = 0.9939 |  |  |  |
|  | **ShMOLLI**  a*R*^2^ = 0.9907 | Diff: 0.0032, NS  95% CI: –0.0217 to 0.0395 |  |  |
|  | **SASHA**  a*R*^2^ = 0.9991 | **Diff: –0.0052, *P* < 0.001**  95% CI: –0.0290 to 0.0008 | **Diff: –0.0084, *P* < 0.001**  95% CI: –0.0444 to –0.0002 |  |
| **B** | | **1.5T Philips** | | |
|  |  | **MOLLI**  a*R*^2^ = 0. 9962 | **ShMOLLI**  a*R*^2^ = 0. 9939 | **SASHA**  a*R*^2^ = 0.9987 |
| **1.5T Philips** | **MOLLI^+^**  a*R*^2^ = 0.9962 |  |  |  |
|  | **ShMOLLI^++^**  a*R*^2^ = 0.9939*sic.* | Diff: 0.0023, NS  95% CI: –0.0134 to 0.0263 |  |  |
|  | **SASHA**  a*R*^2^ = 0.9987 | Diff: –0.0025, NS  95% CI: –0.0174 to 0.0035 | Diff: -0.0048, NS  95% CI: –0.0286 to 0.0023 |  |
| **C** | | **1.5T Siemens** | | |
|  |  | **MOLLI**  a*R*^2^ = 0.9939 | **ShMOLLI**  a*R*^2^ = 0.9907 | **SASHA**  a*R*^2^ = 0.9991 |
| **1.5T Philips** | **MOLLI^+^**  a*R*^2^ = 0.9962 | Diff: –0.0023, NS  95% CI: –0.0263 to 0.0134 | Diff: –0.0055, NS  95% CI: –0.0416 to 0.0111 | Diff: 0.0029, NS  95% CI: –0.0018 to 0.0178 |
|  | **ShMOLLI**  a*R*^2^ = 0.9939*sic.* | Diff: 0.000, NS  95% CI: –0.0243 to 0.0243 | Diff: –0.0032, NS  95% CI: –0.0395 to 0.0217 | Diff: 0.0052, NS  95% CI: –0.0008 to 0.0290 |
|  | **SASHA**  a*R*^2^ = 0.9987 | Diff: –0.0048, NS  95% CI: –0.0286 to 0.0023 | Diff: –0.0080, NS  95% CI: –0.0440 to 0.0010 | Diff: 0.0004, NS  95% CI: –0.0033 to 0.0056 |

Significant differences between correlations are highlighted in **bold**.

^+^ Philips data for the MOLLI variant 5b(1b)1b(1b)1b that were not fitted by the iterative/data dropping steps in map creation as per ShMOLLI, are not included.

^++^ Using iterative/data dropping steps in map creation as per ShMOLLI.

a*R*^2^ adjusted R-squared against *T*_1_; CI, 95% confidence interval; Diff: difference; GE: General Electric; NS, not significant.

**Supplementary Table 2.** Differences between the correlations of the absolute measurement of *T­_1_* and “reference *T_1_*” (*rT_1_*) for a given sequence at 3T when compared to an alternative sequence on the same platform (**Panels A** and **B**) or to a similar sequence on a different platform (**Panel C**). *rT*_1_ times were obtained by slow inversion recovery at Royal Brompton Hospital. GE and Agilent not included (too few centers).

| **A** |  | **3T Siemens** | |
| --- | --- | --- | --- |
|  |  | **MOLLI**  a*R*^2^ = 0.9958 | **ShMOLLI**  a*R*^2^ = 0.9897 |
| **3T Siemens** | **MOLLI**  a*R*^2^ = 0.9958 |  |  |
|  | **ShMOLLI**  a*R*^2^ = 0.9897 | Diff: 0.0061, NS  95% CI: ­–0.0123 to 0.0460 |  |
|  | **SASHA**  a*R*^2^ = 0.9997 | **Diff: –0.0039, *P* < 0.001**  95% CI: –0.0203 to –0.0003 | **Diff: –0.0100,** ***P* < 0.001**  95% CI: –0.0497 to –0.0017 |
| **B** | | **3T Philips** | |
|  |  | **MOLLI^+^**  a*R*^2^ = 0. 9976 | **SASHA**  a*R*^2^ = 0.9894 |
| **3T Philips** | **MOLLI^+^**  a*R*^2^ = 0.9976 |  |  |
|  | **SASHA**  a*R*^2^ = 0.9894 | Diff: 0.0082, NS  95% CI: –0.0045 to 0.0491 |  |
| **C** | | **3T Siemens** | |
|  |  | **MOLLI**  a*R*^2^ = 0.9958 | **SASHA**  a*R*^2^ = 0.9997 |
| **3T Philips** | **MOLLI^+^**  a*R*^2^ = 0.9976 | Diff: –0.0018, NS  95% CI: –0.0183 to 0.0082 | Diff: 0.0021, NS  95% CI: –0.0002 to 0.0115 |
|  | **SASHA**  a*R*^2^ = 0.9894 | Diff: 0.0064, NS  95% CI: –0.0121 to 0.0474 | **Diff: 0.0103, *P* < 0.001**  95% CI: 0.0018 to 0.0511 |

Significant differences between correlations are highlighted in bold.

^+^ Philips data for the MOLLI variant 5b(1b)1b(1b)1b that were not fitted by the iterative/data dropping steps in map creation as per ShMOLLI, are not included.

Abbreviations as in **Supplementary Table 1**.

**Supplementary Table 3.** Temperature correction ($\beta$) according to the formula: $T\text{1} =Intercept +\left( \beta*\left[ Temperature -21 \right] \right)$, per tube by field-strength, derived from linear regression equations using multi-center T1MES data. In line with results from parallel temperature-controlled experiments conducted at the US National Institute of Standards and Technology laboratory, these data indicate that short-*T*_1_ tubes (modelling post-GBCA myocardium and blood) are more stable with temperature than very long-*T*_1_ tubes (native blood) where *T*_1_ increased more significantly with temperature (as would be expected from the basic *T*_1_ temperature sensitivity of water). Tubes are listed in the order they appear at the time of scanning (top > bottom; left > right, see inset image). Temperature-corrected (21°C) “reference standard” *T*_1_ values (*rT*_1_) are additionally provided for each tube as well as the % difference these corrections represent relative to the *rT*_1_.

| **Tube # *[1.5T / 3T ID]***  **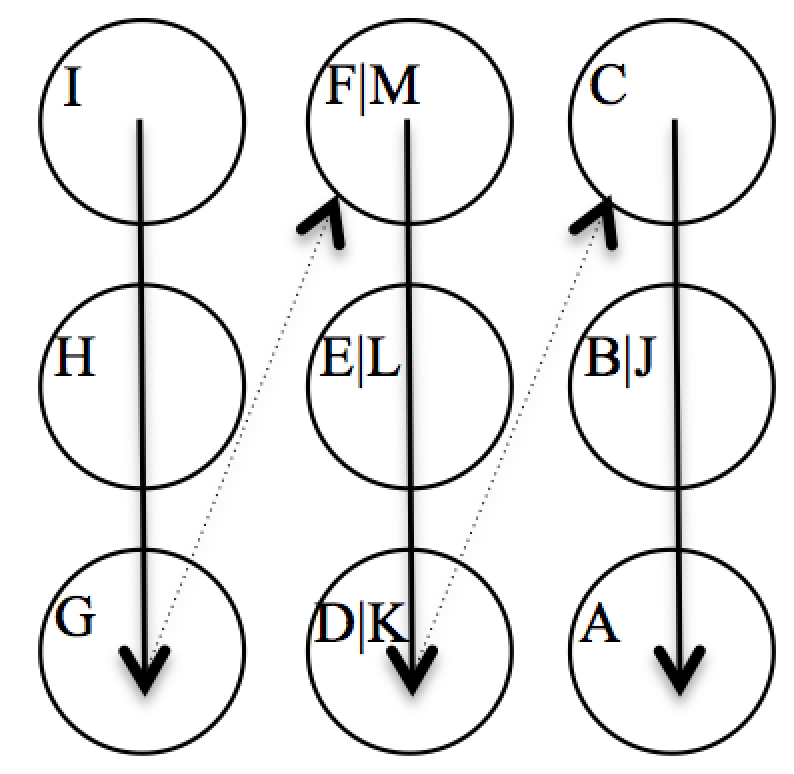** | **Temperature correction (in ms) per °C increase** | |
| --- | --- | --- |
|  | **1.5T Temperature correction**  **[*% change relative to rT_1_ at 21°C at 2 years*]** | **3T Temperature correction**  **[*% change relative to rT_1_ at 21°C at 2 years*]** |
| “Medium” post-GBCA myocardium *[I / I]* | -1.03  [*0.23 % of 440 ms*] | 0.15  [*0.03 % of 429 ms*] |
| “Long” post-GBCA myocardium *[H / H]* | -0.59  [*0.10 % of* *572 ms*] | 0.94  [*0.17 % of 563 ms*] |
| “Short” post-GBCA myocardium *[G / G]* | -0.94  [*0.31 % of* *300 ms*] | -0.21  [*0.07 % of 297 ms*] |
| “Medium” native myocardium *[F / M]* | 4.97  [*0.44 % of 1121 ms*] | 9.62  [*0.74 % of 1298 ms*] |
| “Long” native myocardium *[E / L]* | 9.97  [*0.71 % of 1401 ms*] | 14.02  [*0.90 % of 1552 ms*] |
| “Short” native myocardium *[D / K]* | 2.10  [*0.25 % of 825 ms*] | 5.80  [*0.56 % of 1031 ms*] |
| “Long” post-GBCA blood *[C / C]* | -0.85  [*0.18 % of 468 ms*] | 1.73  [*0.38 % of 456 ms*] |
| “Normal” native blood *[B / J]* | 12.46  [*0.80 % of 1562 ms*] | 23.91  [*1.24 % of 1935 ms*] |
| “Short” post-GBCA blood *[A / A]* | -0.80  [*0.31 % of 259 ms*] | -0.07  [*0.03 % of 251 ms*] |

GBCA = gadolinium-based contrast agent; ID = tube identity.

**Supplementary Table 4.** List of T1MES collaborators at enrollment (in alphabetical order by surname, excluding co-authors). The list includes center-specific principal investigators but also key members of their local teams (e.g. fellows, radiographers) known to have contributed voluntary time to this program. We apologise for any omissions.

| Trevor | Ahearn |
| --- | --- |
| Ibitisam | Alanizi |
| Aiman | Alnajjar |
| Andrew | Arai |
| Svein | Are Vatnehol |
| Shanat | Baig |
| Gavin | Bainbridge |
| Patricia | Bandettini |
| Jan | Bogaert |
| David | Broadbent |
| Greg | Brown |
| Jennifer | Bryant |
| Emma | Caffery |
| Antonia | Camporeale |
| Jyh-Wen | Chai |
| Yi-Pin | Chang |
| Mark | Chapman |
| Ken | Cheng |
| Dante | Chiappino |
| Amedeo | Chiribiri |
| Kelvin | Chow |
| David | Clark |
| Isabelle | Cloutier |
| David | Corcoran |
| Ben | Costello |
| Gary | Cowin |
| Deep | Dastidar |
| Paul | De Bruin |
| Nicky | Edwards |
| Pershina | Ekaterina |
| Andris | Ellims |
| Caius | Fabian |
| Paul | Fergusson |
| Juliano | Fernandes |
| Luciana | Fioravante |
| Alison | Fletcher |
| James | Foley |
| John | Foster |
| Yaron | Fridman |
| Matthias | Friedrich |
| Graham | Galloway |
| Oliver | Geier |
| Suzanne | Gommers |
| Matt | Graham-Brown |
| Gaurav | Gulsin |
| Vedant | Gupta |
| Tarik | Hafyane |
| Peter | Hardy |
| James | Hare |
| Taigang | He |
| John | Hoover |
| Einar | Hopp |
| David | Horne |
| Leah | Iles |
| Baljit | Jagpal |
| Amy | Jenkinson |
| Andreas | Kammerlander |
| Rebecca | Kozor |
| Anna | Kydd |
| Andre | Lagerche |
| Chirs | Lawton |
| Ying-Hsang | Liao |
| Ayers | Lisa |
| Chia-Ying | Liu |
| Grethe | Løvland |
| Boyang | Lui |
| Chris | Mancini |
| Kenneth | Mangion |
| Nicola | Martini |
| Julia | Mascherbauer |
| Gerry | McCann |
| Louise | McGrath |
| Simon | McGuirk |
| Elena | Mershina |
| Michael | Mikolaj |
| Chris | Miller |
| Stuart | Moir |
| Amanda | Murphy |
| Harmen | Mulder |
| Vivek | Muthurangu |
| Josephine | Naish |
| Arthur | Nasis |
| Bac | Nguyen |
| Sabrina | Nordin |
| Vanessa | Orchard |
| Malou | Paiman |
| Dilveer | Panesar |
| John | Payne |
| Patrizia | Pedrotti |
| Charles | Peebles |
| Dudley | Pennell |
| Marcel | Prothmann |
| Kathy | Puntil |
| Aleksandra | Radjenovic |
| Claudio | Rapezzi |
| Samuli | Rauhalammi |
| Jonathan | Rodrigues |
| Alberto | Roghi |
| Kathrine | Ryden Suther |
| Dan | Sado |
| Erik | Schelbert |
| Matthias | Schmitt |
| Bernhard | Schnackenburg |
| Johannes | Schueler |
| Jeanette | Schulz-Menger |
| Francesco | Secchi |
| Frank | Seifert |
| Michael | Sellenger |
| Giancarlo | Serafini |
| Arash | Seratnahaei |
| Joanne | Simpson |
| Vincent | Sorrell |
| Bruce | Spottiswoode |
| Fabian | Springer |
| Jennifer | Steeden |
| Tracey | Steedman |
| Dan | Swarbrick |
| Andrew M | Taylor |
| Christoph | Tillmanns |
| Maryse | Tremblay |
| Rob | Van Der Geest |
| Pieternel | Van Der Tol |
| Moriel | Vandsburger |
| Niels | Vejlstrup |
| Florian | Von-Knobelsdorff |
| Rick | Wage |
| Andrea | Wiethoff |
| Timothy C | Wong |
| Rosie | Wooward |
| Jo | Wormleighton |
| Yi-Ying | Wu |
| Albert | Yen |
|  |  |
|  |  |
|  |  |
|  |  |
|  |  |
|  |  |
|  |  |
|  |  |
|  |  |
|  |  |
|  |  |
|  |  |
|  |  |
|  |  |
|  |  |
|  |  |
|  |  |
|  |  |
|  |  |
|  |  |
|  |  |
|  |  |
|  |  |
|  |  |
|  |  |

**Supplementary Table 5.** Temperature-adjusted (normalized to 21°C) mean CoV (%) of *T*_1_ per post-GBCA tube by the various post-GBCA *T*_1_ mapping sequences at 1.5T according to sequence and prototype/product variant.

| **Summary Table of *x̅* CoV of *T*_1_ (%) Across 5 Tubes** | | | **^§^Order Of Tube IDs Follows Their Orientation In The Scanned Bottle** | | | | |
| --- | --- | --- | --- | --- | --- | --- | --- |
| Siemens | ShMOLLI 5b(1b)1b(1b)1b [1041B] | **0.21** | **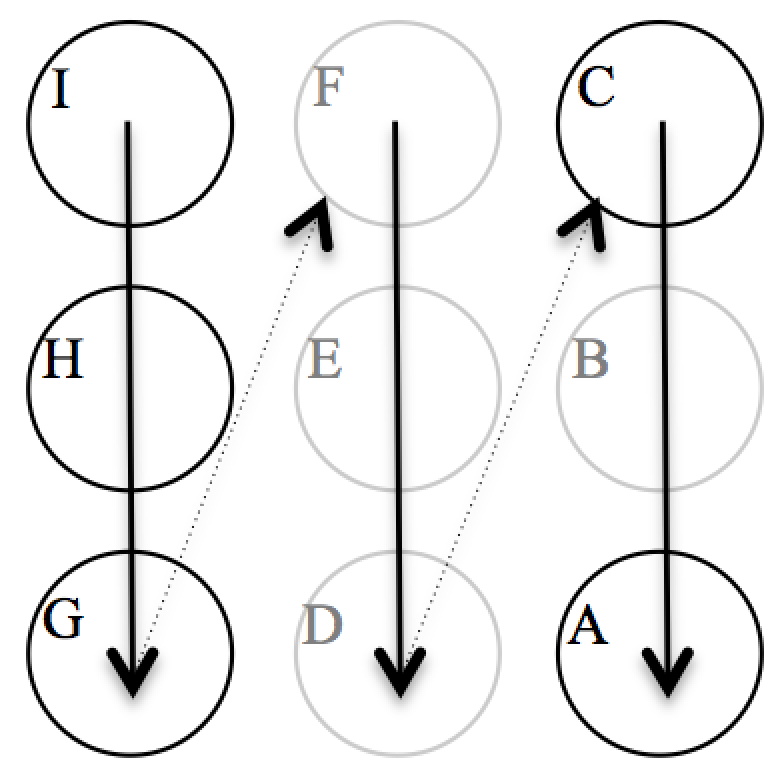** | | | | |
| Siemens | MOLLI 4s(1s)3s(1s)2s [448B] | **0.26** |  |  |  |  |  |
| Philips | MOLLI 4b(1s)3b(1s)2b | **0.59** |  |  |  |  |  |
| Siemens | SASHA | **0.68** |  |  |  |  |  |
| Philips | ShMOLLI 5b(1b)1b(1b)1b^++^ | **0.77** |  |  |  |  |  |
| GE | MOLLI 5b(1b)1b(1b)1b^+^ | **1.05** |  |  |  |  |  |
| GE | SMART | **5.18** |  |  |  |  |  |
|  |  |  |  |  | | | |
| **Platform** | **Sequence [Prototype, #]** | | ***x̅’* CoV (%) of *T*_1_ According To Post-GBCA Tube ID^§^** [Tube *rT*_1_ by slow IR] | | | | |
|  |  |  | **I** [430ms] | **H** [562ms] | **G** [300ms] | **C** [458ms] | **A** [255ms] |
| **Siemens** | MOLLI MyoMaps Product 4s(1s)3s(1s)2s [*5*] | | 0.26 | 0.38 | 0.40 | 0.27 | 0.37 |
|  | MOLLI 4b(1s)3b(1s)2b [448, *3*] | | 0.25 | 0.24 | 0.36 | 0.20 | 0.39 |
|  | MOLLI 4b(1s)3b(1s)2b [448B, *2*] | | 0.50 | 0.50 | 0.54 | 0.53 | 0.64 |
|  | MOLLI 4b(1s)3b(1s)2b [780, *1*] | | 0.36 | 0.46 | 0.57 | 0.48 | 0.46 |
|  | MOLLI 4b(1s)3b(1s)2b [780B, *3*] | | 0.48 | 0.47 | 0.67 | 0.53 | 0.90 |
|  | MOLLI 4s(1s)3s(1s)2s [448, *1*] | | 1.07 | 1.03 | 1.47 | 0.92 | 1.11 |
|  | MOLLI 4s(1s)3s(1s)2s [448B, *1*] * | | 0.33 | 0.20 | 0.28 | 0.30 | 0.20 |
|  | MOLLI 4s(1s)3s(1s)2s [780B, *2*] | | 0.37 | 0.52 | 0.36 | 0.30 | 0.43 |
|  | MOLLI 4s(1s)3s(1s)2s [1041, *1*] | | 0.39 | 0.65 | 0.59 | 0.98 | 0.51 |
|  | ShMOLLI 5b(1b)1b(1b)1b [448, *1*] | | 0.66 | 0.74 | 0.50 | 0.60 | 0.60 |
|  | ShMOLLI 5b(1b)1b(1b)1b [448C, *1*] | | 0.41 | 0.55 | 0.56 | 0.46 | 0.52 |
|  | ShMOLLI 5b(1b)1b(1b)1b [780B, *3*] | | 0.45 | 1.31 | 0.43 | 0.39 | 0.36 |
|  | ShMOLLI 5b(1b)1b(1b)1b [1048, *1*] | | 0.34 | 0.45 | 0.33 | 0.41 | 0.30 |
|  | ShMOLLI 5b(1b)1b(1b)1b [1041B, *1*] * | | 0.14 | 0.34 | 0.20 | 0.27 | 0.12 |
|  | SASHA [*2*] | | 0.65 | 0.47 | 1.05 | 0.55 | 0.71 |
| **Philips** | MOLLI CardiacQuant Product 4s(1s)3s(1s)2s [*2*] | | 0.40 | 0.77 | 0.74 | 0.39 | 0.91 |
|  | MOLLI 4b(1s)3b(1s)2b [*1*] * | | 0.38 | 1.12 | 0.54 | 0.44 | 0.48 |
|  | MOLLI 4s(1s)3s(1s)2s [*3*] | | 0.65 | 1.02 | 0.97 | 0.67 | 1.20 |
|  | ShMOLLI 5b(1b)1b(1b)1b^++^ [*1*] | | 0.96 | 0.78 | 0.71 | 0.65 | 0.76 |
| **GE** | MOLLI 5b(1b)1b(1b)1b^+^ [*1*] | | 1.69 | 0.85 | 0.61 | 0.42 | 1.67 |
|  | SMART [*1*] | | 3.16 | 0.80 | 1.97 | 3.22 | 16.77 |

* Denotes the *T*_1_ mapping sequence|software combination with lowest overall CoV% for a given vendor where multiples exist.

# Denotes the number of different magnets submitting that particular sequence from which the average CoVs were derived.

^++^ Using iterative/data dropping steps in map creation as per ShMOLLI.

^+^ In the absence of iterative/data dropping steps in map creation, i.e. not ShMOLLI.

*x̅* = average CoV across the 5 post-GBCA tubes for a given sequence.

*x̅’* = where more than one sequence type was submitted, individual CoVs were then averaged to derive *x̅’* CoV; while for single sequence submissions *x̅’* CoV is from the global mean T1±SD for that one sequence.

Higher CoVs (>1%, see **Methods Part 3–*T_1_ repeatability***) are highlighted in red. Native tubes are not shown here as their data are reported separately in relation to native sequences, in **Table 1** (main text).

MOLLI/ShMOLLI protocol nosology has the number of inversions per experiment as the total count of numbers outside brackets, image cycles are outside brackets, pause cycles are within brackets, and cycle lengths defined in terms of either heart beats (b) or seconds (s).

*x̅* = average CoV across the 5 post-GBCA tubes for a given sequence; CoV = coefficient of variation; GE = General Electric; MOLLI = modified Look-Locker inversion recovery; *rT*_1_ = “reference” slow inversion recovery *T*_1_; SASHA = saturation-recovery single-shot acquisition; ShMOLLI = shortened MOLLI; SMART = saturation method using adaptive recovery times for cardiac *T*_1_ mapping; T = Tesla. Other abbreviations as in **Supplementary Table 4**.

**Supplementary Table 6.** Temperature-adjusted (normalized to 21°C) mean CoV (%) of *T*_1_ per post-GBCA tube by the various post-GBCA *T*_1_ mapping sequences at 3T according to sequence and prototype/product variant.

|  | | |  |  | | | | |
| --- | --- | --- | --- | --- | --- | --- | --- | --- |
|  | | |  |  | | | | |
| **Summary Table of *x̅* CoV of *T*_1_ (%) Across 5 Tubes** | | | **^§^Order Of Tube IDs Follows Their Orientation In The Scanned Bottle** | | | | | |
| **Siemens** | MOLLI 4b(1s)3b(1s)2b [448B] | **0.10** | **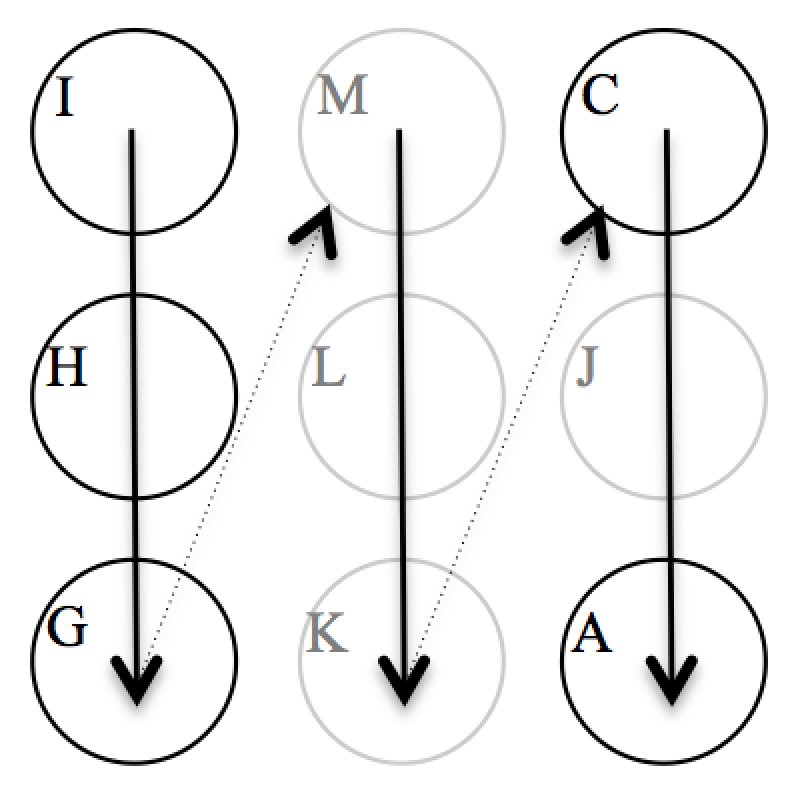** | | | | | |
| **Siemens** | ShMOLLI 5b(1b)1b(1b)1b [780C] | **0.28** |  |  |  |  |  |  |
| **Philips** | MOLLI 4s(1s)3s(1s)2s | **0.30** |  |  |  |  |  |  |
| **Siemens** | SASHA | **0.49** |  |  |  |  |  |  |
| **GE** | SMART | **5.24** |  |  |  |  |  |  |
|  |  |  |  |  |  |  |  |  |
| **Platform** | **Sequence [Prototype, #]** | | ***x̅’* CoV (%) of *T*_1_ According To Native Tube ID^§^** [Tube *rT*_1_ by slow IR] | | | | | |
|  |  |  | **I** [430ms] | | **H** [562ms] | **G** [300ms] | **C** [458ms] | **A** [255ms] |
| **Siemens** | MOLLI MyoMaps Product 4s(1s)3s(1s)2s [*4*] | | 0.23 | | 0.24 | 0.26 | 0.40 | 0.33 |
|  | MOLLI 4b(1s)3b(1s)2b [448B, *1*] * | | 0.05 | | 0.02 | 0.07 | 0.11 | 0.21 |
|  | MOLLI 4b(1s)3b(1s)2b [780B, *1*] | | 0.37 | | 0.46 | 0.31 | 0.31 | 0.29 |
|  | MOLLI 4s(1s)3s(1s)2s [780B, *1*] | | 0.17 | | 0.20 | 0.29 | 0.17 | 0.77 |
|  | ShMOLLI 5b(1b)1b(1b)1b [780B, *2*] | | 0.39 | | 0.43 | 0.31 | 0.41 | 0.15 |
|  | ShMOLLI 5b(1b)1b(1b)1b [780C, *1*] * | | 0.27 | | 0.33 | 0.33 | 0.26 | 0.22 |
|  | SASHA [*1*] | | 0.41 | | 0.41 | 0.38 | 0.22 | 1.01 |
| **Philips** | MOLLI 4b(1s)3b(1s)2b [*1*] | | 0.32 | | 0.38 | 2.42 | 0.28 | 2.53 |
|  | MOLLI 4s(1s)3s(1s)2s [*6*] * | | 0.27 | | 0.21 | 0.62 | 0.24 | 0.18 |
|  | MOLLI 5b(1b)1b(1b)1b^+^ [*2*] | | 4.59 | | 0.46 | 1.68 | 3.40 | 2.35 |
| **GE** | SMART [*1*] | | 4.66 | | 1.18 | 6.24 | 5.10 | 9.01 |

* Denotes the *T*_1_ mapping sequence|software combination with lowest overall CoV% for a given vendor where multiples exist.

# Denotes the number of different magnets submitting that particular sequence from which the average CoVs were derived.

^+^ In the absence of iterative/data dropping steps in map creation, i.e. not ShMOLLI.

*x̅* = average CoV across the 5 post-GBCA tubes for a given sequence.

*x̅’* = where more than one sequence type was submitted, individual CoVs were then averaged to derive *x̅’* CoV; while for single sequence submissions *x̅’* CoV is from the global mean T1±SD for that one sequence.

Less favourable CoVs (>2%, see **Methods Part 3–*T_1_ repeatability***) are highlighted in red. Native tubes are not shown here as their data are reported separately in relation to native sequences, in **Table 2** (main text).

Abbreviations as in **Supplementary Tables 4** and **5**.

**Supplementary Table 7.** Fixed effect and variance component parameter estimates from final model ‘**A5**’ at 1.5T.

| **Random effects** | **Variance** | **Standard deviation** |  |
| --- | --- | --- | --- |
| Random intercept variance (ID) | 2547.2 | 50.5 |  |
| Residual variance | 539.5 | 23.23 |  |
| **Fixed effects** | ***β* Coef. estimate** | ***β* Coef. standard error** | **95% Confidence intervals** |
| Intercept | 607.1 | 52.1 | 492.4 – 721.9 |
| Sequence­_MOLLI 3s(3s)3s(3s)5s | 187.6 | 97.0 | -7.7 – 383.9 |
| Sequence_MOLLI 3s(3s)5s | -31.3 | 21.2 | -73.0 – 10. 3 |
| Sequence_MOLLI 5b(3s)3b | 38.7 | 8.9 | 21.3 – 56.1 |
| Sequence_MOLLI 5b(3s)3b(3s)2b | 156.2 | 9.1 | 138.3 –174.1 |
| Sequence_MOLLI 5b(3s)5b | 46.2 | 16.7 | 13.4 – 79.0 |
| Sequence_MOLLI 5s(3s)3s | 69.1 | 11.7 | 46.0 – 92.1 |
| Sequence_MOLLI MyoMaps Product | 61.0 | 11.7 | 38.1 – 83.9 |
| Sequence_SASHA | 167.3 | 10.9 | 145.9 – 188.8 |
| Sequence_ShMOLLI | -32.3 | 6.0 | -44.1 – -20.4 |
| Sequence_SMART | 455.6 | 72.7 | 308.7 – 602.4 |
| Software_R3.2 | 452.3 | 72.4 | 305.9 – 598. 7 |
| Software_R4.1 | 522.3 | 105.4 | 311.1 – 734.5 |
| Software_R5.1 | 404.9 | 72.9 | 257.8 – 552.1 |
| Software_R5.2 | 496.1 | 74.4 | 346.5 – 646.4 |
| Software_VB17 | 385.1 | 92.7 | 198.1 – 572.0 |
| Software_VD13 | 467.5 | 101.9 | 275.9 – 659.6 |
| Software_VE11 | 487.5 | 101.9 | 282.0 – 693.1 |
| Model_Aera | -90.6 | 84.9 | -262.1 – 80.5 |
| Model_Avanto | 16.0 | 73.8 | -132.8 – 164.8 |
| Model_Ingenia | -161.8 | 74.2 | -311.7 – -12.8 |
| Sequence MOLLI 5b(3s)3b : Software R3.2 | -43.7 | 8.8 | -61.1 – -26.5 |
| Sequence SASHA : Software R4.1 | -161.8 | 23.9 | -208.6 – -115.0 |
| Sequence ShMOLLI : Software R4.1 | -109.7 | 22.0 | -153.0 – -66.4 |
| Sequence MOLLI 5s(3s)3s : Software R5.1 | -54.5 | 73.0 | -202.0 – 92.7 |
| Sequence SASHA : Software R5.1 | -103.1 | 14.2 | -131.1 – -75.2 |
| Sequence MOLLI 5b(3s)3b : Software VB17 | -6.9 | 12.1 | -30.7 – 16.9 |
| Sequence MOLLI 5s(3s)3s : Software VB17 | -25.8 | 56.5 | -139.8 – 88.0 |
| Sequence MOLLI 5s(3s)3s : Software VD13 | -22.2 | 14.6 | -50.8 – 6.5 |
| Sequence SASHA : Software VD13 | -75.1 | 24.1 | -122.5 – -27.7 |

Coef = coefficient.

**Supplementary Table 8.** Fixed effect and variance component parameter estimates from final model ‘**A3**’ at 3T.

| **Random effects** | **Variance** | **Standard deviation** |  |
| --- | --- | --- | --- |
| Random intercept variance (ID) | 372.4 | 19.3 |  |
| Residual variance | 1089.8 | 33.0 |  |
| **Fixed effects** | ***β* Coef. estimate** | ***β* Coef. standard error** | **95% Confidence intervals** |
| Intercept | 1241.4 | 29.3 | 1183.5 – 1300.6 |
| Sequence: MOLLI 3b(3s)5b | 17.0 | 33.0 | -47.9 – 81.8 |
| Sequence: MOLLI 3s(3s)5s | 38.5 | 23.4 | -9.5 – 87.6 |
| Sequence: MOLLI 5b(3s)3b | -41.2 | 28.7 | -99.4 – 15.2 |
| Sequence: MOLLI 5s(3s)3s | -45.4 | 39.2 | -122.5 – 32.2 |
| Sequence: MOLLI MyoMaps Product | -58.0 | 31.4 | -121.4 – 4.2 |
| Sequence: SASHA | 36.8 | 27.9 | -19.8 – 91.6 |
| Sequence: ShMOLLI | -114.5 | 26.9 | -169.0 – -61.7 |
| Sequence: SMART | -90.9 | 35.4 | -163.5 – -20.4 |
| Software: R3.2 | -203.0 | 42.2 | -287.6 – -119.7 |
| Software: R5.1 | -105.0 | 27.0 | -159.7 – -52.0 |
| Software: VB17 | -149.3 | 37.0 | -224.7 – -75.9 |
| Software: VB20 | 32.7 | 33.3 | -33.2 – 99.5 |
| Sequence MOLLI 5b(3s)3b : Software R3.2 | 88.1 | 41.7 | 6.2 – 170.6 |
| Sequence SASHA : Software R3.2 | 267.3 | 40.0 | 188.7 – 346.6 |
| Sequence ShMOLLI : Software R3.2 | -48.8 | 39.2 | -125.9 – 29.1 |
| Sequence MOLLI 5s(3s)3s : Software R5.1 | 77.1 | 39.2 | -0.5 – 154.3 |
| Sequence SASHA : Software R5.1 | 77.8 | 29.8 | 19.1 – 137.1 |
| Sequence MOLLI 5b(3s)3b : Software R5.1 | -10.7 | 36.1 | -82.5 – 60.6 |

Abbreviations as in **Supplementary Table 7**.

**Supplementary Table 9.** Temperature-adjusted (normalized to 21°C) mean CoV (%) of ECV by the various native and post-GBCA *T*_1_ mapping sequences at both field strengths according to sequence and prototype/product variant.

| **Platform** | **Native Sequence [Prototype] + Post-GBCA Sequence [Prototype, #1.5T, #3T]** | **CoV (%) of ECV According To Field Strength** | |
| --- | --- | --- | --- |
|  |  | **1.5T** | **3T** |
| **Siemens** | MOLLI MyoMaps Product 5s(3s)3s & MOLLI MyoMaps Product 4s(1s)3s(1s)2s [*5, 4*] | 0.73 | 0.60 |
|  | MOLLI 3b(3s)3b(3s)5b [448] & MOLLI 4b(1s)3b(1s)2b [448, *1, ­–*] | 0.84 | / |
|  | MOLLI 5b(3s)3b [448] & MOLLI 4b(1s)3b(1s)2b [448, *2, –*] | 0.42 * | / |
|  | MOLLI 5b(3s)3b [448B] & MOLLI 4b(1s)3b(1s)2b [448B, *2, 1*] | 0.94 | 0.10 * |
|  | MOLLI 5b(3s)3b [780] & MOLLI 4b(1s)3b(1s)2b [780, *1, –*] | 0.87 | / |
|  | MOLLI 5b(3s)3b [780B] & MOLLI 4b(1s)3b(1s)2b [780B, *3, 1*] | 0.84 | 0.27 |
|  | MOLLI 5s(3s)3s [448] & MOLLI 4s(1s)3s(1s)2s [448, *1, –*] | 2.23 | / |
|  | MOLLI 5s(3s)3s [448B] & MOLLI 4s(1s)3s(1s)2s [448B, *1, –*] | 0.58 | / |
|  | MOLLI 5s(3s)3s [780B] & MOLLI 4s(1s)3s(1s)2s [448B, *2, 1*] | 0.96 | 0.27 |
|  | MOLLI 5s(3s)3s [1041] & MOLLI 4s(1s)3s(1s)2s [1041, *1, –*] | 2.06 | / |
|  | ShMOLLI 5b(1b)1b(1b)1b [448, *1, –*] | 2.06 | / |
|  | ShMOLLI 5b(1b)1b(1b)1b [448C, *1, –*] | 0.67 | / |
|  | ShMOLLI 5b(1b)1b(1b)1b [780B, *3, 2*] | 2.62 | 0.40 * |
|  | ShMOLLI 5b(1b)1b(1b)1b [780C, *–, 1*] | / | 0.40 * *sic.* |
|  | ShMOLLI 5b(1b)1b(1b)1b [1048, *1, –*] | 0.23 * | / |
|  | ShMOLLI 5b(1b)1b(1b)1b [1041B, *1, –*] | 0.75 | / |
|  | SASHA & Post-GBCA SASHA [*2, 1*] | 1.26 | 0.45 |
| **Philips** | MOLLI CardiacQuant Product 5s(3s)3s & MOLLI CardiacQuant Product 4s(1s)3s(1s)2s [*2, –*] | 1.61 * | / |
|  | MOLLI 3b(3s)3b(3s)5b & MOLLI 4b(1s)3b(1s)2b [*1, –*] | 2.82 | / |
|  | MOLLI 5b(3s)3b & MOLLI 4b(1s)3b(1s)2b [*–, 1*] | / | 0.28 * |
|  | MOLLI 5s(3s)3s & MOLLI 4s(1s)3s(1s)2s [*3, 4*] | 1.90 | 2.14 |
|  | MOLLI 5b(1b)1b(1b)1b^+^ [*–, 1*] | / | 2.36 |
|  | ShMOLLI 5b(1b)1b(1b)1b^++^ [448, *1, –*] | 1.82 | / |
| **GE** | MOLLI 5b(1b)1b(1b)1b^+^ [*1,* –] | 6.14 | / |
|  | SMART [*1, 1*] | 5.32 | 6.32 |

Although the TIMES phantom does not have an intracellular and extracellular volume and cannot therefore serve as a reference standard for ECV, we sought to model derived ‘ECV’ data for any center that submitted intra-scan (paired) pre and post-GBCA MOLLI and SASHA sequences, as well as for centers submitting ShMOLLI or SMART T_1_. ECV was derived at 1.5T (***Eq.1***) and 3T (***Eq.2***) arbitrarily assuming a “phantom hematocrit” of 0.425 as previously described[1]:

$$\mathbf{ECV for 1.5T T1MES} =$$

$\left( 1 -hematocrit \right)* \left( \frac{\begin{matrix} \frac{1}{Long post-GBCA myocardium "Tube H"} & - \frac{1}{\text{Medium} native myocardium "Tube F"} \end{matrix}}{\begin{matrix} \frac{1}{Long post-GBCA blood "Tube C"} & - \frac{1}{Normal native blood "Tube B"} \end{matrix}} \right)$ ***Eq.1***

$$\mathbf{ECV for 3T T1MES}=$$

$\left( 1 -hematocrit \right)*\left( \frac{\begin{matrix} \frac{1}{Long post-GBCA myocardium "Tube H"} & - \frac{1}{\text{Medium} native myocardium "Tube M"} \end{matrix}}{\begin{matrix} \frac{1}{Long post-GBCA blood "Tube C"} & - \frac{1}{Normal native blood "Tube J"} \end{matrix}} \right)$***Eq.2***

* Denotes the *T*_1_ mapping sequence|software combination with lowest overall CoV% for a given vendor where multiples exist. MOLLI and SASHA, ECV was only calculated where matched native and post-GBCA sequences had been submitted by participating centers.

_#_ Denotes the number of different magnets at 1.5T and 3T respectively, submitting that particular sequence combination from which the average CoVs were derived.

^++^ Using iterative/data dropping steps in map creation as per ShMOLLI.

^+^ In the absence of iterative/data dropping steps in map creation as per ShMOLLI.

**Supplementary Figure 1.** Plots showing temperature-corrected “reference” *rT*_1_ (**A**) and *rT*_2_ (**B**) data for 3T phantom 30E021 acquired at Physikalisch-Technische Bundesanstalt over a period of 1041 days (64 scans) commencing September 2015. Sequences used for *rT*_1_ and *rT*_2_ were respectively basic single-slice TR=8000ms IRSE (IRSE, 7 TI from 25–4800ms) and single-slice TR=3000ms SE (5 TE from 24–400ms). The phantom was stored, moved, and scanned while resting in a styrofoam box to ensure that the temperatures picked at bottle hull reflected the tube temperature (this setup was based on local investigator preference and differed from that prescribed in the T1MES user manual that was used by all other centers). At scan time the styrofoam box containing phantom 30E021 (**C**) was placed in the head coil (12-channel) of the 3T MAGNETOM Verio scanner (Siemens Healthcare, Erlangen, Germany; software syngo MR B17A). No further thermal insulation was used. Over 2 years, the systematic shift in *rT*_1_ was 0.80% and for *rT*_2_ it was 1.65%.

**
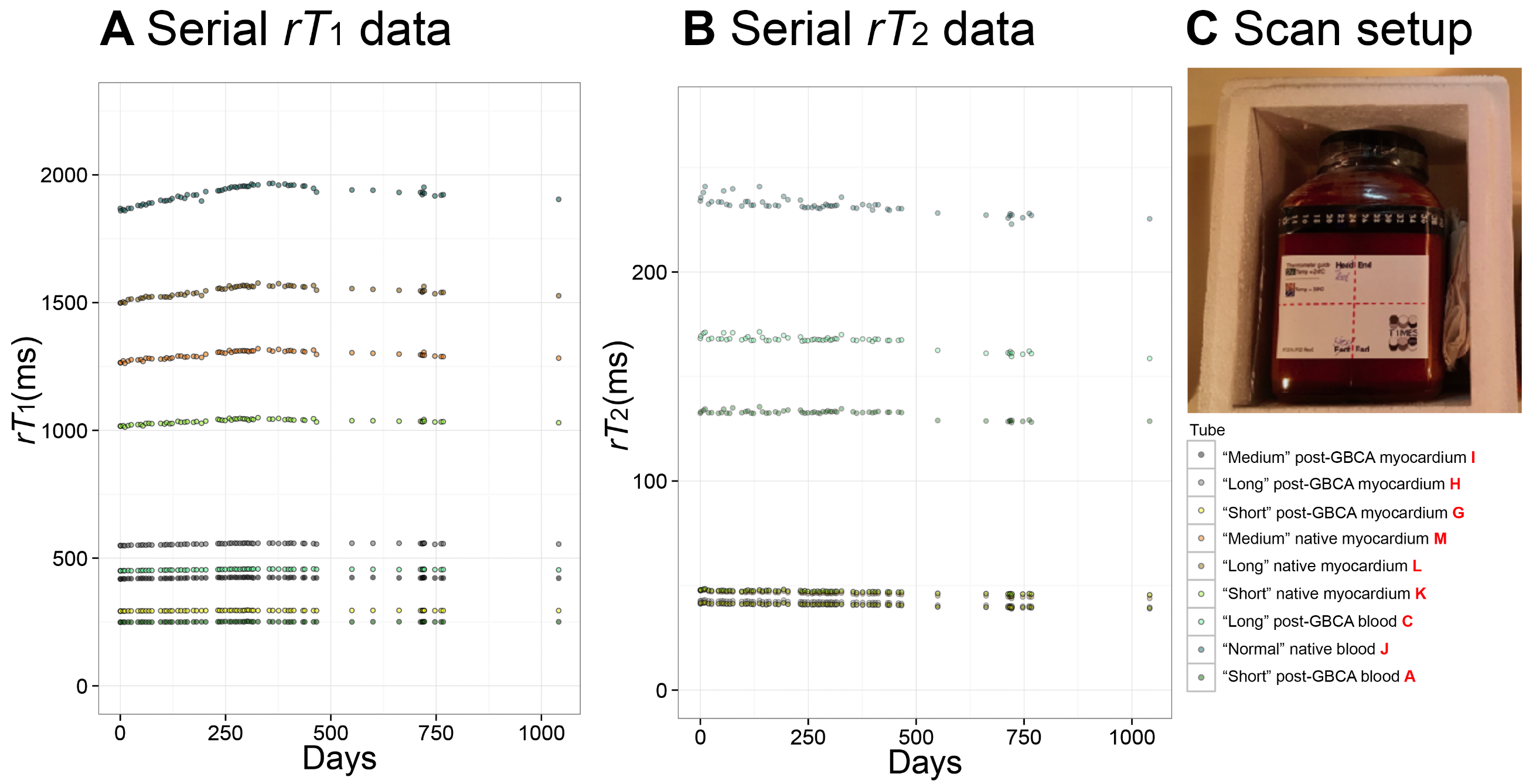
**

**Supplementary Figure 2.** Examples of quality assurance alerts during corelab of early T1MES contributions. A) T1MES scanned with shouldering phantom objects not in accordance with T1MES user manual; B) T1MES not at isocenter due to insufficient supporting material on table, resulting in a –*y* offset; C) T1MES scanned upside down–the brightest (white = longest T_1_) tube “J” (arrow head) should be right but is here left (Philips *T*_1_ map: no color scale bar generated); D) Incorrect acquisition along the tubes’ long axis; E) Sequence reconstruction failure (here ShMOLLI 1.5T Siemens); F) Probably a SSFP stabilisation artefact over tube “J” (white arrow head) by SMART1 mapping on 3T GE scanner.

SSFP = steady-state free precession.


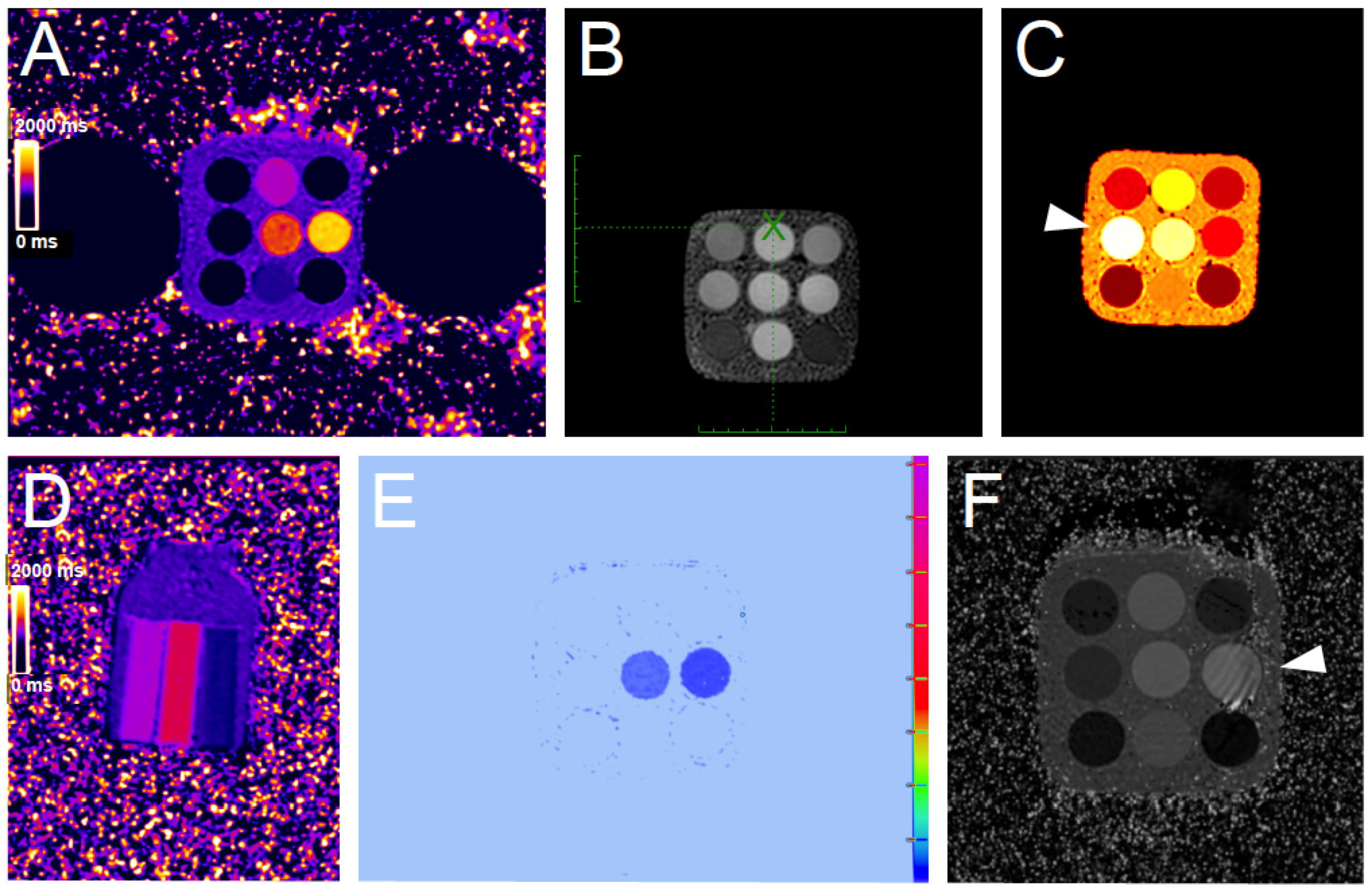


**Supplementary Figure 3.** The relationship between scan day and temperature-unadjusted phantom *T*_1_ times for two centers that submitted duplicate *T*_1_ mapping sequences per scan session plotted as transparent points for each scan session (**A** 15E009: 1.5T Philips Ingenia, R5.1.7SP2, duplicate MOLLI 5s(3s)3s; **B** 30E014: 3T Siemens, MAGNETOM Prisma, VE11, duplicate MOLLI MyoMaps Product). These data suggest that typical short-term thermal noise random jitter is negligible within individual scan sessions (transparent points show excellent overlay).

**
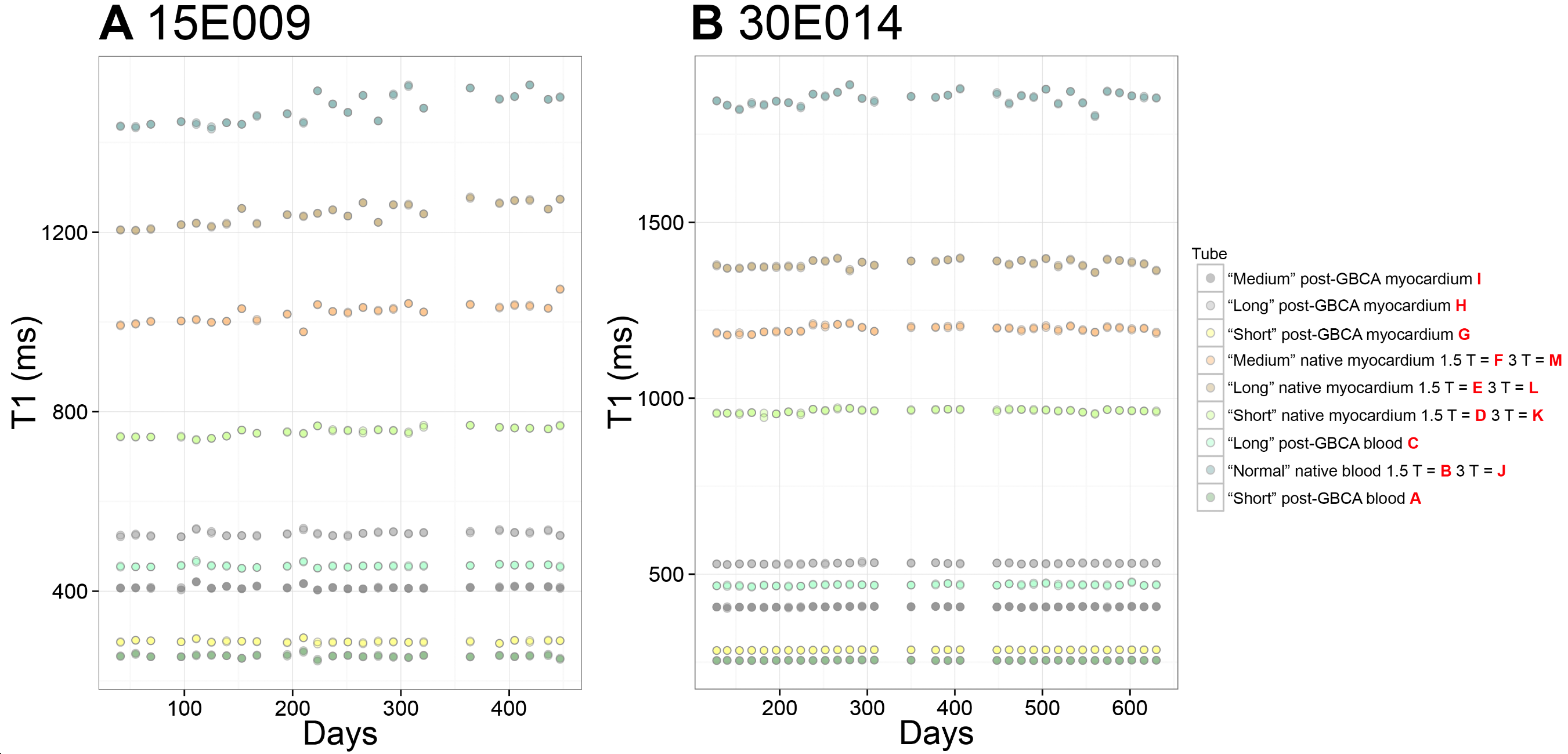
**

**Supplementary Figure 4.** Software upgrades screening results. The relationship between scan day and temperature-unadjusted phantom *T*_1_ times for the “medium” native myocardial *T*_1_ tube (“F” at 1.5T / “M” at 3T) is modeled as piece-wise linear function (horizontal blue lines). DICOM-confirmed potential shift events (software upgrades) are marked by vertical red lines. Here we show models for the 3 T1MES magnets that underwent a software upgrade during the project lifecycle (two Siemens and one GE magnet systems). Software upgrades on the two Siemens systems occurred towards the end of the longitudinal data submission (**A**, 15E031 from VB17A to VE11; **B**, 30E017 from VD13C to VE11) and visually appear not to have caused a significant *T*_1_ shift however differences between pre- and post- linear regression slopes cannot be reliably interpreted due to sparse data [single data point] beyond the upgrade). The software upgrade on GE (**C**, 30E012 from DV25.0 R01 1451.a to DV25.0 R02 1549.b) occurred earlier in the longitudinal data submission compared to the Siemens sites, and based on the difference between pre- and post-linear regression slopes, it counts as a marginally significant *T*_1_ shift event (*P*=0.024).

**
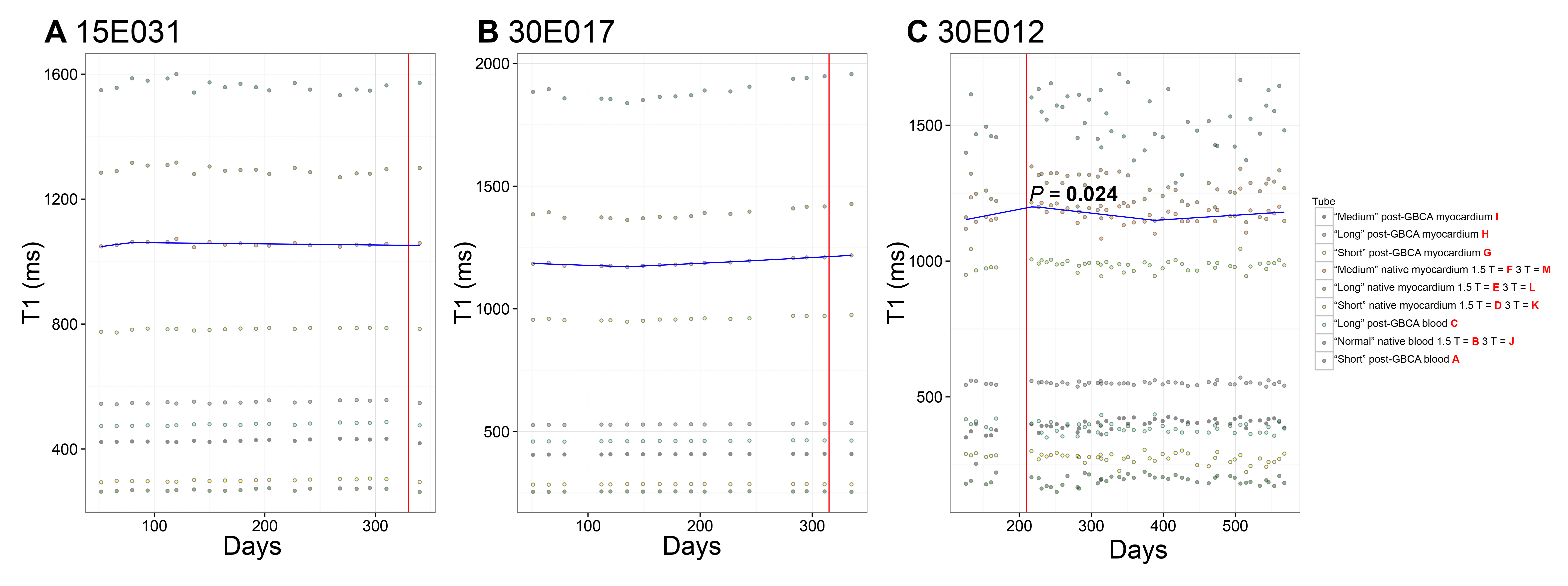
**

**Supplementary Movie 1.** High-resolution imaging of phantom 30E017 at baseline (October 2015, **Left panel**) and at two years post manufacturing (**Right panel**).

**REFERENCES:**

1. Vassiliou VS, Heng EL, Gatehouse PD, Donovan J, Raphael CE, Giri S, Babu-narayan S V, Gatzoulis MA, Pennell DJ, Prasad SK, Firmin DN: **Magnetic resonance imaging phantoms for quality-control of myocardial T1 and ECV mapping: specific formulation, long-term stability and variation with heart rate and temperature**. *J Cardiovasc Magn Reson* 2016:1–12.
